# Simulating photodynamic therapy for the treatment of glioblastoma using Monte Carlo radiative transport

**DOI:** 10.1117/1.JBO.29.2.025001

**Published:** 2024-02-06

**Authors:** Louise Finlayson, Lewis McMillan, Szabolcs Suveges, Douglas Steele, Raluca Eftimie, Dumitru Trucu, Christian Thomas A. Brown, Ewan Eadie, Kismet Hossain-Ibrahim, Kenneth Wood

**Affiliations:** a SUPA, University of St Andrews, School of Physics and Astronomy, St Andrews, United Kingdom; b University of Dundee, Division of Mathematics, Dundee, United Kingdom; c University of Dundee, Medical School, Division Imaging Science and Technology, Dundee, United Kingdom; d Université de Bourgogne Franche-Comté, Laboratoire Mathématiques de Besançon, Besançon, France; e Ninewells Hospital, Photobiology Unit, Dundee, United Kingdom; f University of Dundee, School of Medicine, Division Cellular and Molecular Medicine, Dundee, United Kingdom; g Ninewells Hospital and Medical School, Department of Neurosurgery, Dundee, United Kingdom

**Keywords:** Glioblastoma, photodynamic therapy, photosensitizer protoporphyrin IX, Monte Carlo radiative transport, *in silico*

## Abstract

**Significance:**

Glioblastoma (GBM) is a rare but deadly form of brain tumor with a low median survival rate of 14.6 months, due to its resistance to treatment. An independent simulation of the INtraoperative photoDYnamic therapy for GliOblastoma (INDYGO) trial, a clinical trial aiming to treat the GBM resection cavity with photodynamic therapy (PDT) via a laser coupled balloon device, is demonstrated.

**Aim:**

To develop a framework providing increased understanding for the PDT treatment, its parameters, and their impact on the clinical outcome.

**Approach:**

We use Monte Carlo radiative transport techniques within a computational brain model containing a GBM to simulate light path and PDT effects. Treatment parameters (laser power, photosensitizer concentration, and irradiation time) are considered, as well as PDT’s impact on brain tissue temperature.

**Results:**

The simulation suggests that 39% of post-resection GBM cells are killed at the end of treatment when using the standard INDYGO trial protocol (light fluence = $200\;J/{\mathrm{cm}}^{2}$ at balloon wall) and assuming an initial photosensitizer concentration of 5  μM. Increases in treatment time and light power (light fluence = $400\;J/{\mathrm{cm}}^{2}$ at balloon wall) result in further cell kill but increase brain cell temperature, which potentially affects treatment safety. Increasing the p hotosensitizer concentration produces the most significant increase in cell kill, with 61% of GBM cells killed when doubling concentration to 10  μM and keeping the treatment time and power the same. According to these simulations, the standard trial protocol is reasonably well optimized with improvements in cell kill difficult to achieve without potentially dangerous increases in temperature. To improve treatment outcome, focus should be placed on improving the photosensitizer.

**Conclusions:**

With further development and optimization, the simulation could have potential clinical benefit and be used to help plan and optimize intraoperative PDT treatment for GBM.

## 1 Introduction

Glioblastoma (GBM) brain tumors are a rare occurrence with only 3.19-4.1/100,000 people diagnosed globally every year.^1^ However, due to their aggressive nature and high resistance to treatment, patient prognosis is often poor, with a median overall survival rate of 14.6 months when treated with the current standard of care, which involves resection surgery followed by radiotherapy and chemotherapy.^2^ An increase in the percentage of tumor removed during resection has been shown to correlate with a positive increase in survival time,^3^ and so there is interest in finding safe ways to maximize the resection margin.

One method that has shown some success in this area is fluorescence guided surgery (FGS), which is a method of tumor margin visualization that has recently advanced into GBM surgery with the FDA approval of 5-aminolevulinic acid (5ALA)—a precursor drug to photosensitizer protoporphyrin IX (PpIX).^2^^,^^4^ This success and approval has rekindled interest in extending the treatment and extent of resection by treating any remaining GBM tissue with photodynamic therapy (PDT).^2^

Our research describes the development of a Monte Carlo radiative transport (MCRT) simulation that uses a realistic 3D computational brain model to simulate intraoperative PDT for GBM. The simulation uses the protocol of the recent INtraoperative photoDYnamic therapy for GliOblastoma (INDYGO) trial, a phase I clinical trial of intraoperative PDT for GBM, as well as a mathematical description of PDT to dynamically model the process. It should be noted that this study was performed independent of the INDYGO trial and is simply a simulation that aims to recreate the protocol used. Within the simulation, radiation fluence rate, photobleaching rate, cellular oxygen usage and recovery rate, and singlet oxygen production rate are all taken into account. A singlet oxygen cell kill threshold value is used to predict the percentage of GBM cells remaining at the end of treatment. By changing parameters such as photosensitizer concentration, treatment time, and light power, this percentage is used to compare each protocol’s efficacy to help understand more about the treatment and how it may be optimized. A heat diffusion code, coupled to the MCRT simulation, is used to calculate the temperature of the brain tissue throughout the treatment and the results used to compare the safety of each protocol. It is hoped that our simulation results will help with future development of PDT for the treatment of GBM.

### 1.1 Photodynamic Therapy

Successful PDT comprises of three important factors: light, photosensitizer, and oxygen, which combine to form a toxic environment, resulting in localized cell death. The treatment starts with the administration of a photosensitizer, designed to selectively accumulate within diseased cells. The treatment area is then illuminated with light, the wavelength of which is selected based upon the absorption spectrum of the photosensitizer and the desired depth of effect. This then induces a reaction within the cell, causing toxic singlet oxygen species to form locally, resulting in the cell’s death.^5^

PDT is used and being investigated as a targeted and minimally invasive treatment option for many areas of oncology such as for skin,^6^ head and neck,^7^ bladder,^8^ lung,^9^ and esophageal cancers.^10^ There are several clinical trials, past, present, and future, aiming to explore the potential of PDT for GBM treatment.^2^^,^^11^

### 1.2 INDYGO Trial

The INDYGO trial was a phase I clinical trial that took place at Lille University Hospital in France. The trial aimed to explore the safety and feasibility of expanding the Stupp protocol by treating the surgical resection cavity walls with PDT during surgery. Due to the diffuse nature of GBM, tumor recurrence is inevitable, with most patients seeing recurrence within a few months.^11^ It has been shown that over 80% of these recurrences occur within <2 centimeters of the original resected tumor mass^3^ and so it is postulated that by safely increasing the extent of tumor resection, patient survival time will also be extended. The overall aim was then to safely increase maximal tumor resection via PDT mediated cell kill, leading to a potential improvement in patient survival time.^4^

The trial protocol starts by administering 5-ALA orally to the patient 6 hours before resection surgery. Once the tumor is removed via FGS, an inflatable balloon containing an optical fiber that is coupled to a 635 nm PDT laser is inserted into the resection cavity via a trocar. Once inserted, the balloon is inflated with intralipid diffusing solution until it conforms to the cavity walls. This then allows the PDT treatment light to be delivered evenly around the walls, maximizing the treatment area.^12^

Preliminary trial results published in 2021^4^ found no adverse effects within any treated patient and an improved median survival rate of 23.1 months. The trial has now moved into a phase II trial named DOSINDYGO (DOSe finding for INDYGO) with the purpose of modifying the light fluence delivered, via longer treatment times and larger light powers, to find the optimum light fluence for treatment effect and tolerability.^13^

### 1.3 Monte Carlo Radiative Transport

Although PDT is now a widely used treatment for skin cancer, there remains much to be investigated about its use within the brain, specifically about the mechanisms behind its ability to kill cells. Additionally, it is challenging to monitor the treatment’s progress in real time. This is due, in part, to the fact that PDT monitoring is generally restricted to the surface in the form of fluorescence measurements. While useful, such measurements contain only limited information about the interaction and effects that the light is having below the surface. This lack of access is the reason why computer simulations, such as MCRT, are valuable. By modeling various elements, such as light propagation, energy, and drug distribution, simulations can be used to plan treatments by finding the optimal parameters for each specific scenario. In the case of clinical trials, full studies can be done *in silico* to help refine the planned protocol before any permissions or patients are needed. This could help to reduce trial costs while also increasing patient safety and potentially improving the chances of treatment success.

MCRT is seen as the “gold standard” for radiation transport modeling. Years of technology development and computational advancements have seen it move successfully into the field of medical physics.^14^ MCRT relies on the random sampling of various probability distribution functions to simulate the random walk and interactions of photons through matter. Optical properties, usually based upon experimental measurements, are applied to the model to reproduce the light-interaction behavior of the material involved (e.g., scattering, absorption, and fluorescent emission).

MCRT has become an invaluable tool for treatment planning and dose finding in several areas of radiative medicine including phototherapy, radiographic imaging (e.g., x-ray investigations), and nuclear medicine (e.g., PET imaging).^15^^–^^17^ MCRT has already been used many times to model PDT. For example, it has been used to simulate both standard and daylight PDT treatment with 5-ALA for various skin cancers,^18^^,^^19^ as well as modeling interstitial PDT treatment of gliomas.^20^^,^^21^

## 2 Methods

Monte Carlo radiative transport (MCRT) was used to simulate light propagation through a computational model replicating the INDYGO trial protocol. The code used was adapted from a “blank” MCRT code used to calculate the fluence rate from a specified light source within a voxel grid.^22^ This code was adapted from one originally developed for astronomy.^23^ Furthermore, to calculate the effect of the PDT treatment on the temperature of the surrounding brain tissue, a heat diffusion code, originally developed by McMillan et al.,^22^ was adapted to calculate the time-dependent temperate structure in a smaller 3D section of the voxel grid used in the MCRT code. The MCRT voxel grid had dimensions 5.8  cm×5.4  cm×3.6  cm with 250×231×155  voxels, and the temperature simulation grid had dimensions 3.5  cm×3.5  cm×3.5  cm with 152×152×152  voxels corresponding to a smaller section of the model covering a distance of 2.5 cm across the top of the cavity and at least 1 cm of surrounding brain tissue to the right of this cavity section. The size of the model used within the temperature simulation needed to be reduced due to computational constraints. A validation for the fluence rate obtained by the MCRT code can be found in the Supplementary Material.

### 2.1 Glioblastoma/Brain Model

**Fig. 1:**
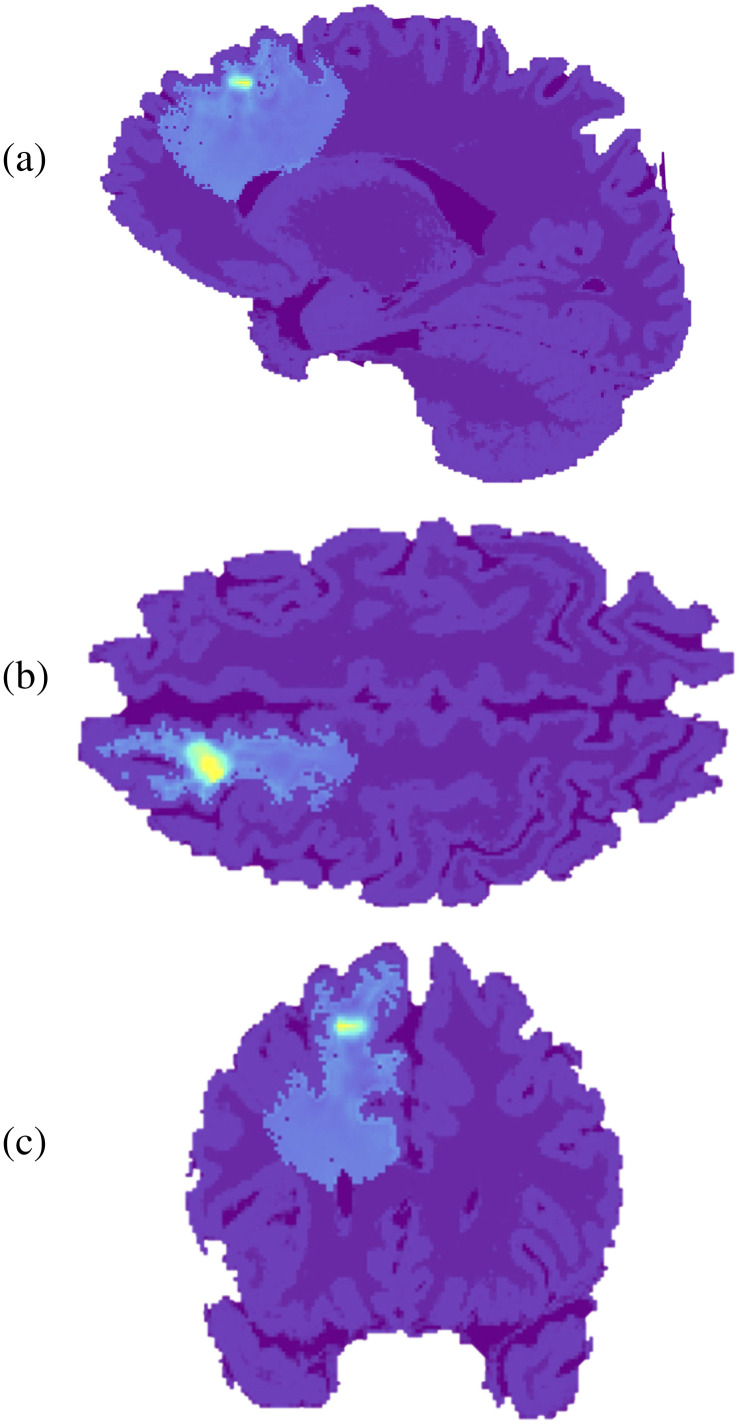
3D computational brain model containing an algorithmically grown GBM, developed at the University of Dundee.^24^ The image shows three 2D slices of the models (a) sagittal plane, (b) coronal plane, and (c) axial plane. The color scale is based on the tissue densities with the darker purple indicating the lower density white matter while the lighter purple shows the higher density gray matter areas. The light blue area is then the GBM tumor, with the yellow area showing the position of the tumor’s necrotic core.

A 3D computational model of a brain containing a GBM was used within the MCRT simulation. The model was developed in collaboration with a neurosurgeon by Suveges et al.^24^ for the purpose of algorithmically modeling the 3D evolution of GBM tumors. The final stage of the model was converted into a format that could be inserted into the MCRT simulation. Figure 1 presents 2D slices of the models sagittal (1a), coronal (1b), and axial (1c) planes before any adaptations were made.

#### 2.1.1 Model adaptations

**Fig. 2:**
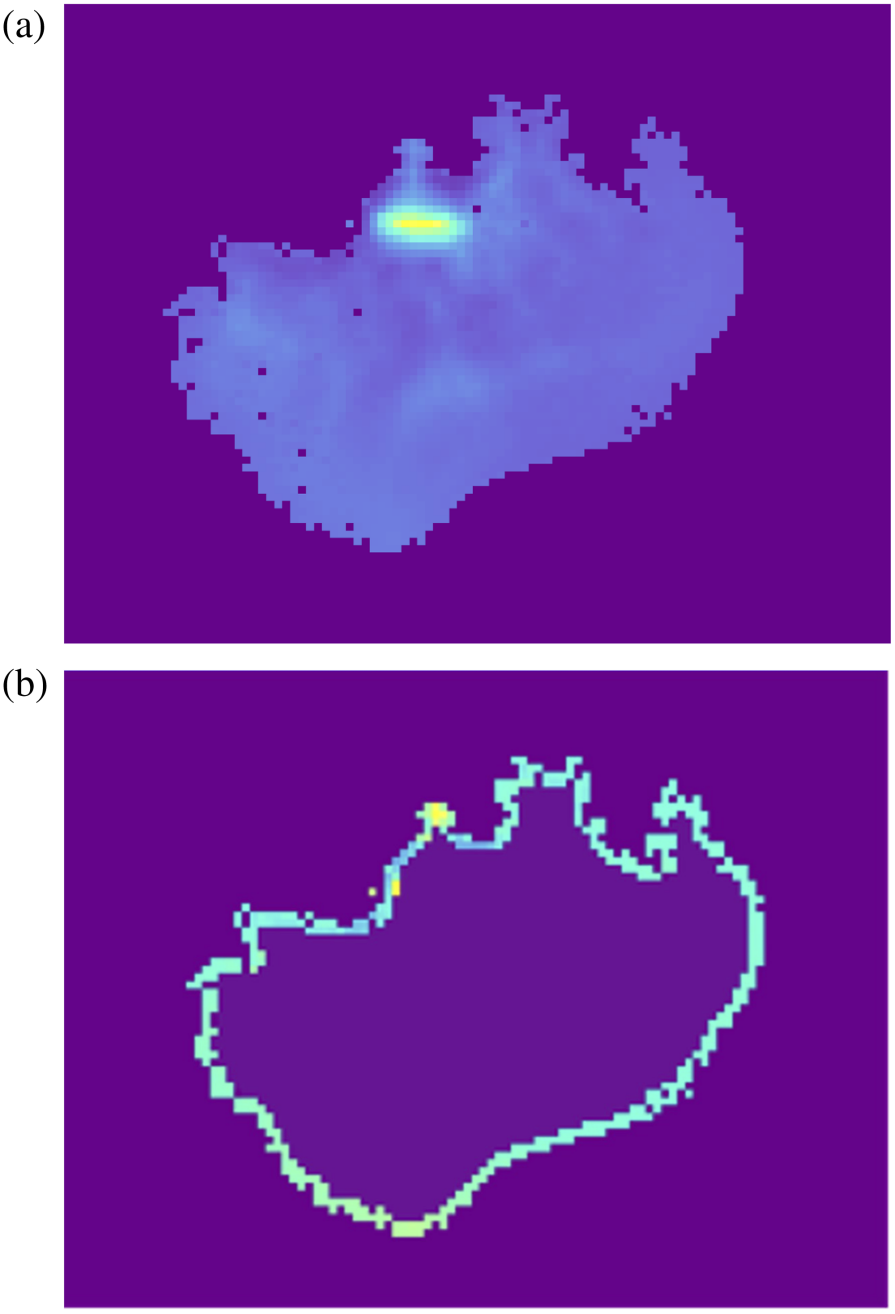
(a) Central slice of isolated simulated GBM (sagittal plane) before simulated resection. (b) Resected slice of GBM tumor. Python programming was used to detect the edges of the tumor with a mask and remove the central part to simulate a resection. As with Fig. 1, the color scale here is density dependent, with the higher density areas of the image marked with yellow and the lowest density areas with purple/blue.

To reproduce the INDYGO trial setup, the brain model had to be adapted. This was done using a Python code. First, the majority of the simulated GBM had to be removed to simulate the tumor resection. A mask was used to detect the tumor edges and remove the central part of the tumor. This left a rim of up to 2 mm unresected tumor remnant, to closely mimic real life clinical cases, where surgeons aim to remove more than 95% of tumor (i.e., rarely achieving complete resection). The result of this is shown in Fig. 2 and checked with a neurosurgeon to accurately reflect real-life clinical scenarios.

Once the resection was complete, a small sphere of tumor was inserted into the top, right hand corner of the cavity, a location where tumor cells are more likely to be missed by the surgeon. This allowed the model to also demonstrate the effect of the simulated protocol on solid tumor (Secs. 2.5.1 and 3.1).

**Fig. 3:**
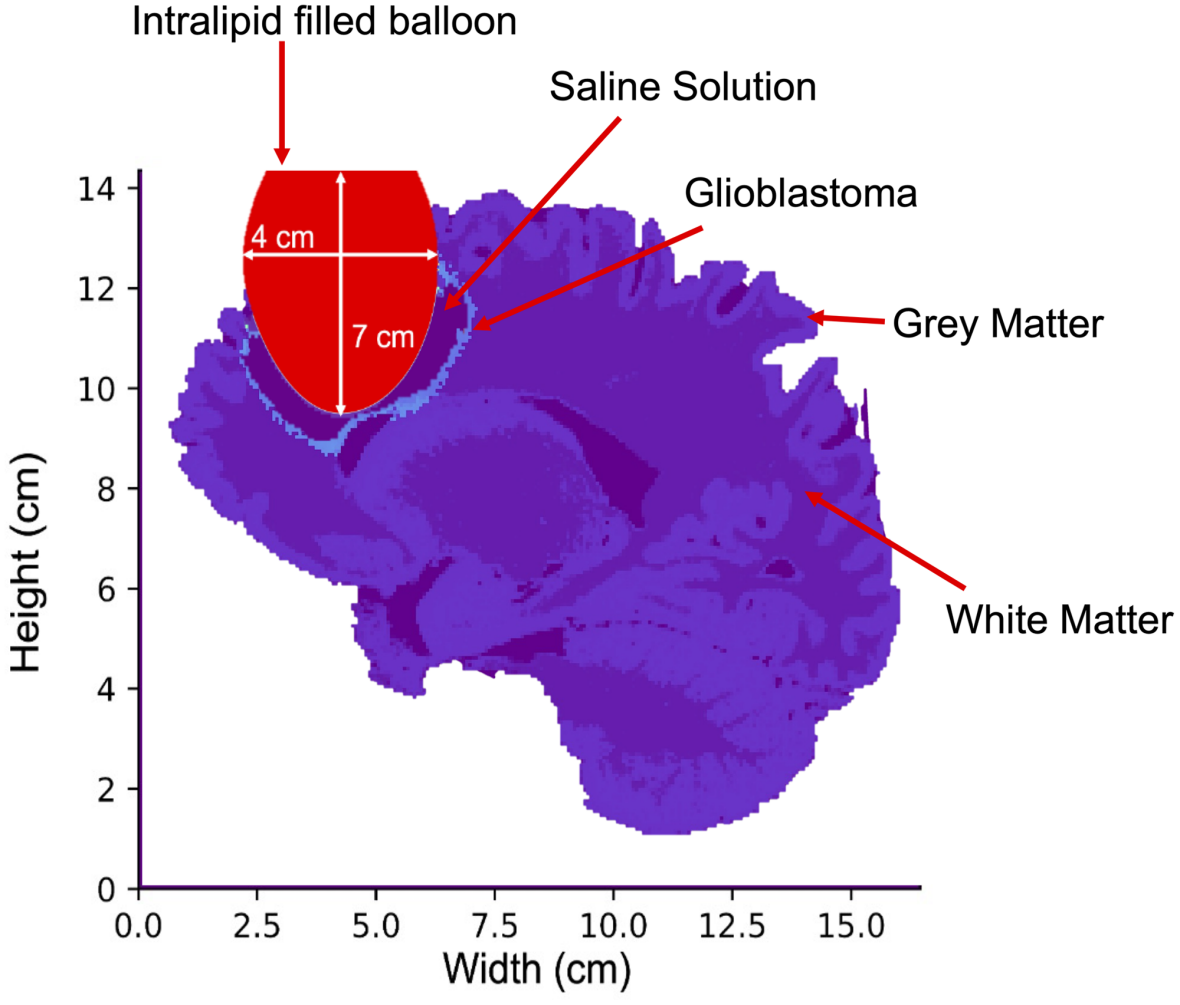
2D slice of final brain model, adapted to reproduce the INDYGO trial setup.

A simulated elliptical balloon with major and minor axis lengths of 7, 4, and 3 cm respectively was then inserted into the resulting cavity. Figure 3 shows a 2D slice of the model with the balloon inserted.

#### 2.1.2 Optical properties

The optical properties within the model vary spatially depending on the biological content within each voxel and are used to help determine the direction and depth that light travels. Each tissue/material type has a unique set of optical properties that consist of the absorption and scattering coefficients ($\mu_{a}$ and $\mu_{s}$), the refractive index (n), and the anisotropy factor (g). Table 1 shows the selected optical properties at 635 nm used for each tissue type. Properties for white matter, gray matter, and GBM tissues are shown, as well as the intralipid scattering fluid that fills the balloon and the saline solution that fills the remaining cavity gaps.

**Table 1:** Optical properties used within the simulation for propagation of 635 nm PDT treatment light.

Tissue/material	Absorption coefficient ($\mu_{a}$) (${\mathrm{cm}}^{-1}$)	Scattering coefficient ($\mu_{s}$) (${\mathrm{cm}}^{-1}$)	Refractive index (n)	Anisotropy factor (g)	References
White matter	0.63	686.0	1.38	0.85	25
Grey matter	0.99	202.0	1.38	0.85	25
GBM	1.3	218.0	1.38	0.85	25
Intralipid solution	0.001	10.0	1.33	0.875	26
Saline solution	0.003	0.003	1.33	0.9	27

The optical properties within each voxel are then determined by the ratio of the tissue types contained within it. Equation (1) shows an example of how each property is calculated where F is the fraction of the voxel that each tissue makes up

$$\mu_{a}=\mu_{a,\text{WhiteMatter}}F_{\text{WhiteMatter}}+\mu_{a,\text{GrayMatter}}F_{\text{GrayMatter}}+\mu_{a,\text{Glioblastoma}}F_{\text{Glioblastoma}}.$$ (1)

The initial concentration of PpIX within each voxel is then determined by Eq. (2) where ${\mathrm{PpIX}}_{0}$ is the chosen initial PpIX concentration in μM for a voxel with 100% GBM tissue

$$C_{\mathrm{PpIX}}=C_{{\mathrm{PpIX}}_{0}}F_{\text{Glioblastoma}}.$$ (2)

The optical properties of PpIX were also taken into account where its absorption coefficient is calculated using Eq. (3)^28^ where $\varepsilon_{630,\mathrm{PpIX}}$ is the PpIX extinction coefficient at 630 nm equal to $0.0265\;{\mathrm{cm}}^{-1}\;{\left(\mu g/\mathrm{ml}\right)}^{-1}$^29^

$$\mu_{a,\mathrm{PpIX}}=C_{\mathrm{PpIX}}\varepsilon_{630,\mathrm{PpIX}}.$$ (3)

As an example, with initial PpIX concentration of 5  μM, the PpIX absorption coefficient within a voxel containing a GBM fraction of 20% would then be

$$\mu_{a,\mathrm{PpIX}}=C_{{\mathrm{PpIX}}_{0}}F_{\text{Glioblastoma}}\varepsilon_{630,\mathrm{PpIX}}=5\times 0.2\times 0.0265\;=0.0265\;{\mathrm{cm}}^{-1}.$$ (4)

### 2.2 Treatment Protocol

To determine the necessary treatment time, an algorithm [Eq. (5)] was developed by the INDYGO trial group to calculate the treatment time (t) in minutes needed for a 2 W light source to achieve a light fluence rate of $200\;J\;{\mathrm{cm}}^{-2}$ at the balloon wall. The algorithm was based on the injected volume of intralipid ($V_{\text{intralipid}}$) needed to cause the balloon to fill the resection cavity^12^

$$t(\min)=0.1176V_{\text{intralipid}}(\mathrm{ml})+3.4276.$$ (5)

The volume of balloon used in the simulation was $44\;{\mathrm{cm}}^{3}$ and for simplicity it was assumed that it would be completely filled with intralipid, resulting in an intralipid volume of 44 ml. Using Eq. (5), this resulted in a calculated treatment time of 8.6 min. While the resulting fluence rate at the balloon wall varied with location, it was within range of the expected value of $200\;J\;{\mathrm{cm}}^{-2}$ (see results in Fig. 5).

To aid triplet oxygen recovery during PDT treatment, the trial protocol also involves fractionating (switching on and off for set periods) the treatment light. For each treatment within the trial, the irradiation time is split into five equal fractions with a rest period of two minutes between each.^13^ This protocol was modeled within our simulations by splitting the treatment time into five equal fractions. At the end of each fraction, the light is switched off for two minutes by setting the fluence rate to zero. This produces a total simulated treatment time of 16.6 min.

An initial PpIX concentration of 5  μM was chosen as the standard value used throughout this study. This was selected based on the range of PpIX concentrations measured by Johansson et al.^30^ in GBM tissues resected during FGS.

### 2.3 PDT Calculations

The simulation uses an algorithm developed by Wang et al.^31^ to model the process of PDT. The algorithm assumes that singlet oxygen production is the main vehicle of malignant cell kill during PDT. Based on the fluence rate within each voxel, calculated using MCRT, the algorithm calculates the photosensitizer concentration, triplet oxygen concentration, and singlet oxygen concentration. This allows it to account for the light fluence reaching each voxel as well as photosensitizer photobleaching and triplet oxygen depletion. The simulation is time dependent and the calculations are repeated within a loop for every second of the selected treatment time. A validation for the PDT results obtained by the MCRT code using Wang et al.’s algorithm can be found in the Supplementary Material.

#### 2.3.1 Photosensitizer concentration

The concentration of photosensitizer, in this case PpIX, within each voxel is calculated first using Eq. (6).^31^ All parameter definitions and values for the PDT calculations can be found in Table 2

δS0(i,j,k)δt+ξσΨ(i,j,k)(S0(i,j,k)+ϱ)[O32(i,j,k)]O32(i,j,k)+βS0(i,j,k)=0. (6)

**Table 2:** Parameter definitions and values for all PDT calculations.

Symbol	Definition	Value/units	References
$S_{0}$	Photosensitizer concentration	$\mu M,t_{0}=\text{varied}$	31
O32	Triplet oxygen concentration	$\mu M,t_{0}=38\;\mu M$	32 and 33^*^
[O12]rx	Apparent reacted singlet oxygen concentration	$\mu M,t_{0}=0\;\mu M$	31
Ψ	Fluence rate	$\mathrm{mW}\;{\mathrm{cm}}^{-3}$	—
t	Time	s	—
ξ	Triplet oxygen consumption rate as a function of photosensitizer concentration and fluence rate. (Before any photobleaching has taken place and assuming an infinite supply of triplet oxygen)	$3.7\times 10^{-3}\;{\mathrm{cm}}^{2}\;{\mathrm{mW}}^{-1}\;s^{-1}$	31
ρ	Low concentration correction	33 μM	31
σ	The ratio of the probability that a singlet oxygen molecule will react with the ground state photosensitizer and the probability that it will react with the cell	$9\times 10^{-5}\;\mu M^{-1}$	31
β	The ratio of the decay rate of the triplet state photosensitizer to ground state and the rate that triplet oxygen quenches the triplet state photosensitizer	11.9 μM	31
Φ	Maximum rate of oxygen perfusion	$21.6\;\mu M\;s^{-1}$	32
$\upsilon_{z}$	Blood flow velocity	$200\;\mu M\;s^{-1}$	32
$R_{c}$	Capillary radius	2.5 μm	34
$R_{t}$	Radius of Krogh tissue cylinder (Half the distance between two capillaries)	30 μm	35
$l_{z}$	Length of capillary	200 μm	32,34
$q_{0}$	Maximum metabolic consumption rate	$26.3\;\mu M\;s^{-1}$	36–38^**^
[O12]rx,threshold	Singlet oxygen threshold concentration	560 μM	39

#### 2.3.2 Triplet oxygen concentration

The triplet or cellular oxygen concentration is then calculated using the following equation:^31^

δO23(i,j,k)δt+(ξΨ(i,j,k)S0(i,j,k)O23(i,j,k)+β)O23(i,j,k)−Φ(t)(1−O23(i,j,k)O23(i,j,k)t=0)=0, (7)

where Φ(t) is the maximum rate of triplet oxygen perfusion. This takes into account both the metabolic consumption and replenishment of triplet oxygen and is calculated using the following equations:^40^^,^^40^^,^^32^

$$\Phi (t)=\Phi_{0}\frac{0.99t^{\prime 4}+1.09t^{\prime 3}+0.05t^{\prime 2}+0.18t^{\prime }+0.32}{t^{\prime 4}+1.16t^{\prime 3}+0.18t^{\prime 2}+0.24t^{\prime }+0.31},$$ (8)

$$t^{\prime }=\frac{t-750}{632.1},$$ (9)

$$\Phi_{0}=\frac{1200\upsilon_{z}R_{c}(R_{c}+\frac{100^{2}+q_{0}^{2}}{50^{2}-q_{0}^{2}})}{l_{z}{\left(R_{t}+4.2\right)}^{2}}.$$ (10)

#### 2.3.3 Singlet oxygen concentration

Finally, the singlet oxygen concentration is calculated using the following equation:^31^

δ[O21(i,j,k)]rxδt−ξΨ(i,j,k)S0(i,j,k)[O23(i,j,k)]O23(i,j,k)+β=0. (11)

At the end of each time step, the singlet oxygen concentration within each voxel is checked and those that have reached the cell kill threshold of 560  μM are marked as having reached the singlet oxygen threshold and are assumed dead. The threshold value of 560  μM was determined by Zhu et al.^39^ when performing *in vivo* measurements in mouse tumor models.

* O23(i,j,k) at time t=0 is set using the equation:

O23(i,j,k)t=0=αPtiO2, (12)

where α is the triplet oxygen solubility in tissue = 1.295  μM/mmHg^32^ and $P{\mathrm{tiO}}_{2}$ is the partial pressure of triplet oxygen in the brain = 30 mmHg.^33^

** This factor was inserted into Eq. (7) under the assumption that metabolic oxygen consumption is significant in the brain within the context of the simulation. A value for this in the required units of $\mu {\mathrm{Ms}}^{-1}$ was produced using the following equation:

$$q_{0,\text{cerebral}}(\mu M\;s^{-1})=q_{0,\text{skin}}(\mu M\;s^{-1})\frac{q_{0,\text{cerebral}}(100\;g^{-1}\;\min^{-1})}{q_{0,\text{skin}}(100\;g^{-1}\;\min^{-1})}.$$ (13)

The cerebral metabolic rate of oxygen consumption according to Patel et al.^36^ and Rink et al.^37^ is $3.5\;\mathrm{ml}\;100\;g^{-1}\;\min^{-1}$. Rink et al.^37^ also states that the metabolic rate of oxygen consumption in the skin is $0.2\;\mathrm{ml}\;100\;g^{-1}\;\min^{-1}$ and the metabolic rate of oxygen consumption in the skin used by Lopez et al.^38^ is $1.5\;\mu M\;s^{-1}$. By making the assumption that $0.2\;\mathrm{ml}\;100\;g^{-1}\;\min^{-1}$ and $1.5\;\mu M\;s^{-1}$ are then equivalent, Eq. (13) uses the ratio of cerebral metabolic oxygen consumption to skin metabolic oxygen consumption to calculate a value for cerebral oxygen consumption in $\mu M\;s^{-1}$

$$q_{0}=1.5\;\mu M\;s^{-1}\frac{3.5\;\mathrm{ml}\;100\;g^{-1}\;\min^{-1}}{0.2\;\mathrm{ml}\;100\;g^{-1}\;\min^{-1}}=26.3\;\mu M\;s^{-1}.$$ (14)

### 2.4 Heat Diffusion Code for Temperature Calculation

The heat diffusion code uses a finite difference method to solve the standard heat equation. A full explanation of the base code is provided by McMillan et al.^22^ The simplest one-dimensional form of the heat equation is described by the following equation:

$$\rho c_{p}\frac{\partial T}{\partial t}=\Delta \cdot (\kappa \Delta T)+\overset{˙}{q},$$ (15)

where ρ is the density ($\mathrm{kg}\;m^{-3}$), $c_{p}$ is the specific heat capacity (${\mathrm{JK}}^{-1}$), κ is the thermal conductivity (${\mathrm{Wm}}^{-1}\;K^{-1}$), T is the temperature at a specific time and position (K), and $\dot{q}$ is the source and sink term at a specific time and position (${\mathrm{Wm}}^{-3}$).

Using Eq. (15), the change in temperature within each voxel, dependent on the energy absorbed and transferred to surroundings, is calculated at each time step. $\dot{q}$ accounts for the external heat sources and sinks. The PDT light source is assumed to be the main source of energy while heat loss due to conduction into the surrounding medium is assumed to be the only sink. The initial temperature of the brain tissue is assumed to be 37°C and everything else is assumed to be room temperature at 22°C. To account for metabolic temperature regulation, boundary conditions are set at the edges facing brain matter to stop the temperature dropping below 37°C. To begin with, the simulation is run for 5 min with the PDT light switched off to allow the initial temperatures to equalize without any input heat.

The power of the light source was changed using the fluence rate grid from the relevant MCRT simulation. It was assumed that the optical properties do not change throughout the treatment time. It was also assumed that the fluence rate does not vary significantly with PpIX concentration due to its relatively low absorption coefficient compared to brain tissue and so only one fluence rate grid was needed for each light power tested.^41^

### 2.5 Parameters Tested

To help increase understanding of the various parameters involved in PDT, such as light power, photosensitizer concentration and treatment time, and their individual effects on the treatment outcome, a range of different protocols were run and results compared to the standard protocol described in Sec. 2.2.

#### 2.5.1 Light penetration and cell kill depth

**Fig. 4:**
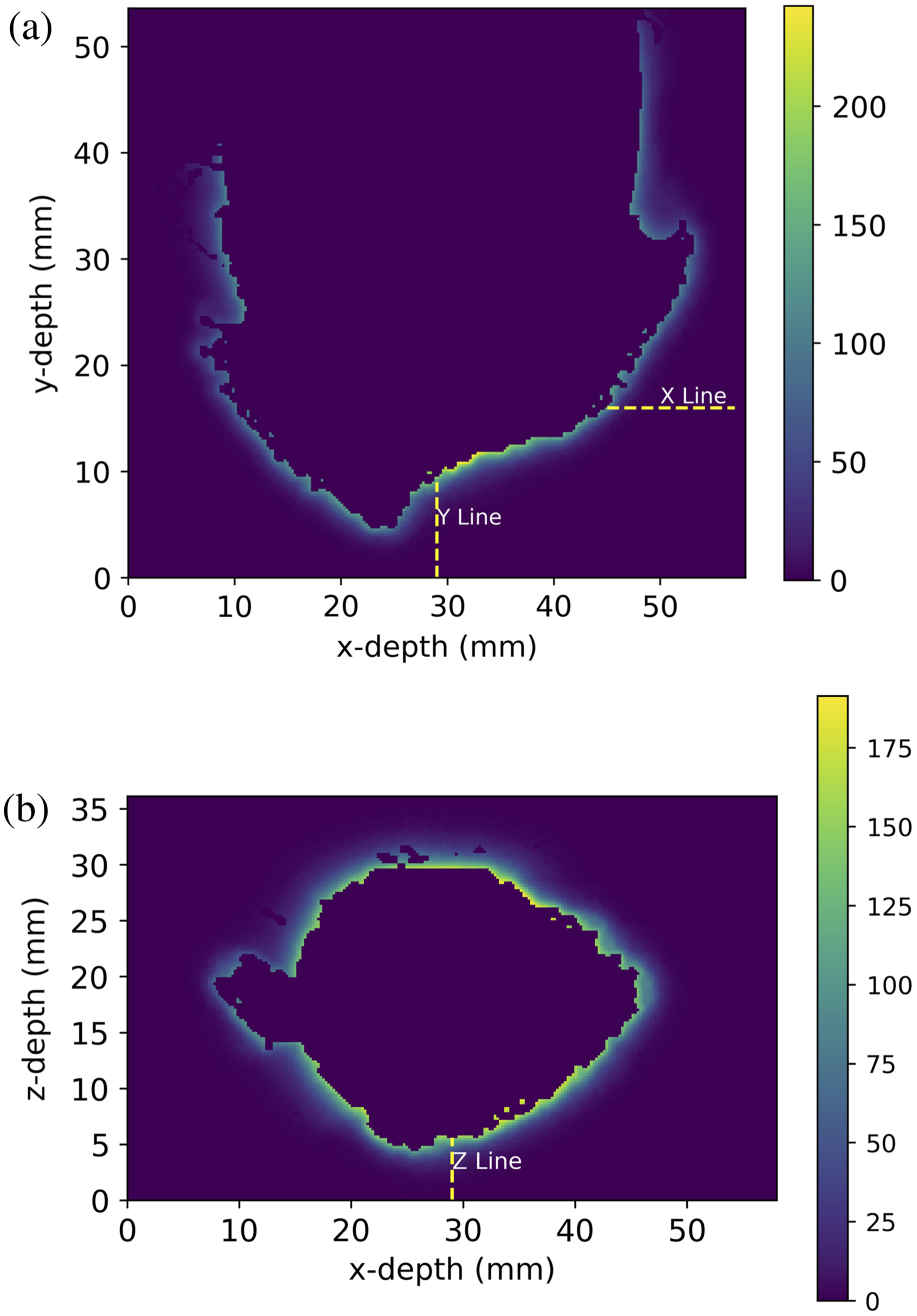
Images showing the light fluence ($J\;{\mathrm{cm}}^{-2}$) from a 2 W laser delivered over 8.6 minutes, indicated by the colour bar, of a central slice of the tumor resection cavity. (a) A slice in the x-y plane and indicates the x and y lines used in data collection. (b) A slice in the x-z plane and indicates the z line used for data collection.

First, to test the depth of cell kill possible using the standard protocol and an initial photosensitizer concentration of 5  μM, the penetration depth of light into the brain and the resulting singlet oxygen concentration was compared for a single point along each of the x, y, and z axes shown in Figs. 4(a) and 4(b). The depth of cell kill into solid tumor using the standard protocol was also investigated by examining the maximum depth into the added tumor sphere that the singlet oxygen cell kill threshold is reached.

#### 2.5.2 Parameter concentration with depth

To demonstrate how the concentrations of PpIX, triplet oxygen and singlet oxygen as well as the temperature at single points in the simulation grid change with depth from the resection cavity wall during the standard protocol treatment, each was plotted over the treatment time for a depth of 0, 1, and 2 mm along the x-line shown in Fig. 4(a).

#### 2.5.3 Photosensitizer concentration

The standard protocol was run for four different initial PpIX concentrations of 1  μM, 3  μM, 5  μM, and 10  μM, similar to the range of photosensitizer concentration values measured in resected GBM tissues by Johansson et al.^30^ The percentage of GBM cells remaining after each second of the treatment time was calculated for each case.

#### 2.5.4 Oxygen depletion

To explore the effect that cellular oxygen depletion has on the treatment outcome, the standard protocol was run using a fixed oxygen concentration of 38  μM [Eq. (12)]. To do this, oxygen depletion was neglected by changing Eqs. (7)–(16)

$$\frac{\delta^{3}O_{2}(i,j,k)}{\delta t}=0.$$ (16)

The purpose of fractionating the treatment light source is to aid cellular oxygen recovery by pausing the PDT treatment to reduce the oxygen usage rate and allow tissue oxygen levels to increase.^13^ The difference between constant and depleted oxygen without light source fractionation was therefore explored by removing the fractionation breaks and instead just keeping the light source on constantly for 8.6 min, delivering the same total amount of energy as the standard, fractionated protocol. The results were again compared by looking at the fraction of GBM cells remaining over the treatment time.

#### 2.5.5 Fractionation

Next, the difference that treatment light fractionation makes to the standard protocol outcome was tested. This was done by running the simulation for the standard protocol time of 16.6 min, but keeping the light on for the full time, neglecting the fractionation breaks. The percentage of GBM cells remaining over the treatment time was then compared to that of the standard protocol with light fractionation. The difference that neglecting fractionation breaks makes to the temperature of the brain tissue was also investigated.

#### 2.5.6 Light fluence

The difference in treatment efficacy by doubling the light fluence was then explored, first by doubling the treatment time, then by doubling the light power. To double the treatment time, the standard protocol was run but with a treatment time of 17.2 min plus 8 min of fractionation breaks making the total time 26.6 min. The resulting GBM cell kill was then compared to the protocol using the standard time. To double the light power, the standard protocol was run using a 4 W light source and its GBM cell kill fraction also compared to the standard protocols. The temperature difference in both cases was also explored.

#### 2.5.7 Cell kill threshold

For the standard simulation, the singlet oxygen concentration threshold for cell kill was chosen to be 560  μM based on values found in literature.^39^ However, it is likely that this value varies with different parameters such as the specific tissue and photosensitizer type and a fully accurate value can then only be obtained by direct measurement. To explore the difference that this threshold value makes to the calculated treatment outcome, the standard protocol was run with the cell kill threshold value varied by ±25% and the percentage of GBM cells remaining over time compared.

## 3 Results

### 3.1 Light Penetration and Cell Kill Depth

**Fig. 5:**
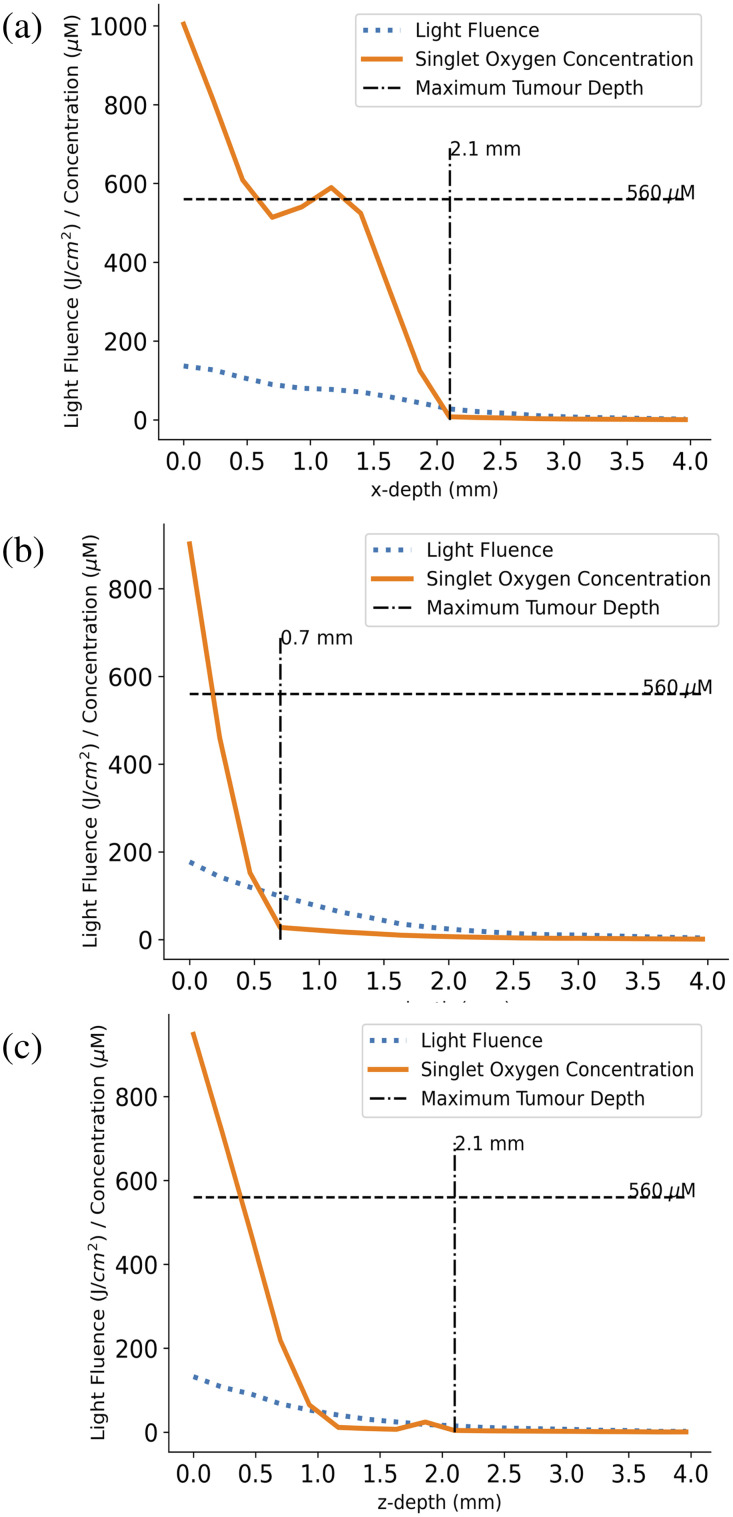
Plots comparing the delivered light fluence and produced singlet oxygen concentration over depth at three points in the x (a), y (b), and z (c) directions shown in Figs. 4(a) and 4(b) at the end of the standard treatment (8.6 min + 8 min of fractionation breaks, 2 W light source, 5  μM initial PpIX concentration). The horizontal dashed lines show the standard threshold singlet oxygen concentration value needed for GBM cell kill, whereas the vertical dashed lines show the maximum depth of GBM cells along these points (2.1 mm in the x and z directions and 0.7 mm in the y direction). The bumps in the singlet oxygen concentration along the x and z directions are caused by the heterogeneous densities of GBM within the tissue, causing variations in the PpIX concentration and resulting singlet oxygen production. Note: variations also occur with differing levels of uptake of PpIX by GBM cells. The plots indicate that while some light appears to reach all GBM cells along these lines, not enough singlet oxygen is being produced at larger depths to cause cell death.

The light fluence and singlet oxygen concentration at the end of the standard treatment protocol along the x, y, and z lines indicated in Fig. 4 are shown in Fig. 5. The vertical dashed lines indicate the maximum depth of GBM cells along these lines. By comparing this to the blue dotted lines indicating the light fluence, we can see that some level of light appears to be reaching all of the tumor cells along each line. However, if we then compare the maximum tumor cell line to the orange solid line indicating singlet oxygen concentration, we can see that the cell kill threshold dose (indicated by the horizontal dashed line) is consistently at a shallower depth suggesting that, using the standard protocol, the singlet oxygen concentration does not get high enough to kill all GBM cells throughout the region of investigation.

**Fig. 6:**
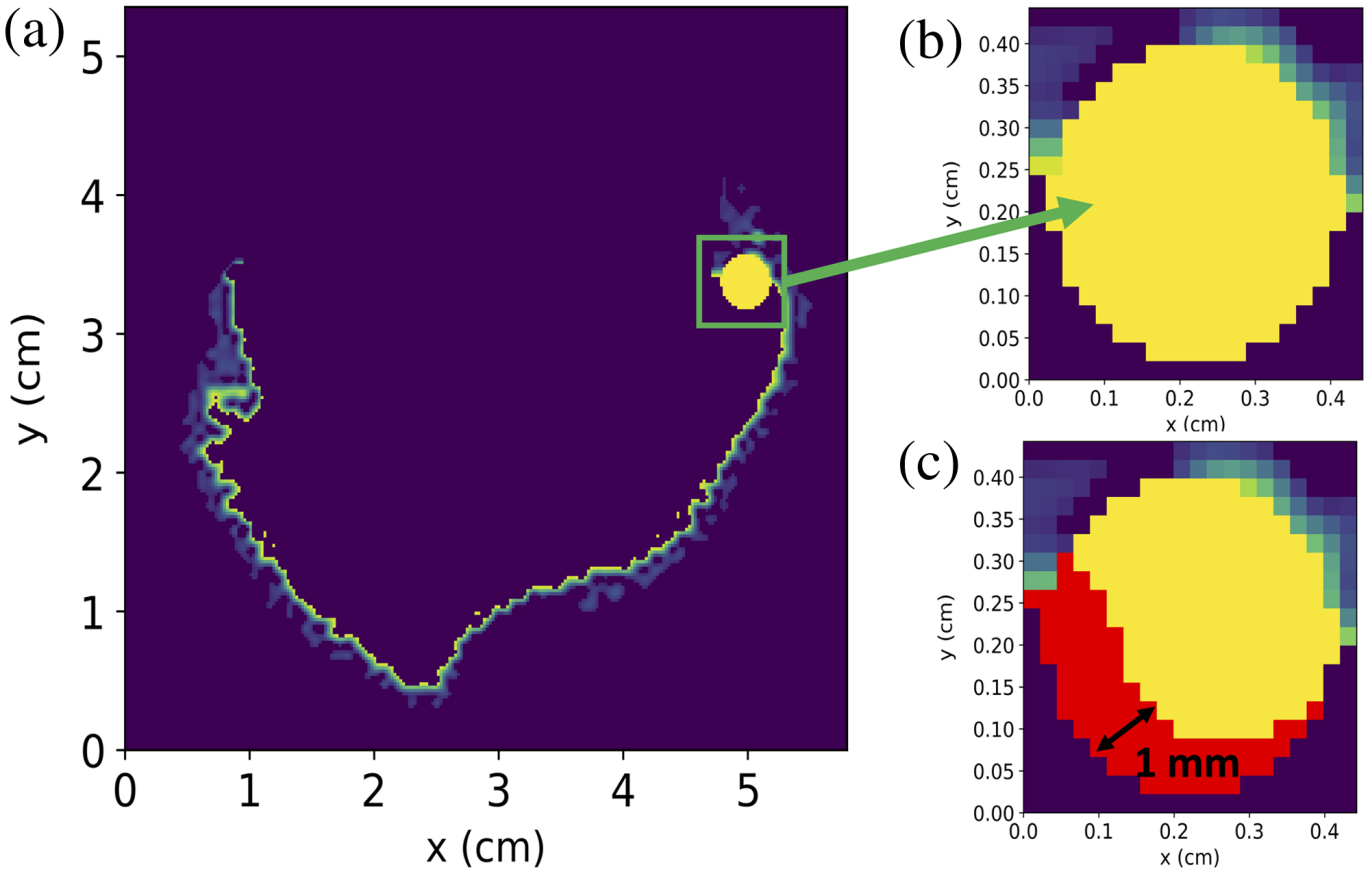
(a) Image showing post-resection and pre-PDT tumor margin with the added sphere of tumor cells in the top right corner of the resection cavity. (b) Zoomed image of the tumor sphere. (c) Image indicating cell kill of the tumor sphere at the end of the standard protocol. The red areas indicate voxels where the singlet oxygen concentration reaches the cell kill threshold, obtaining a cell kill depth of 1 mm.

Figure 6(a) shows an image of the remaining tumor cells around the cavity wall after resection but before PDT. Once the standard protocol is complete, the red areas of Fig. 6(c) indicates the portion of the solid tumor sphere that has reached the singlet oxygen cell kill threshold. We can see that a potential cell kill depth of around 1 mm is obtained.

### 3.2 Tissue Temperature

**Fig. 7:**
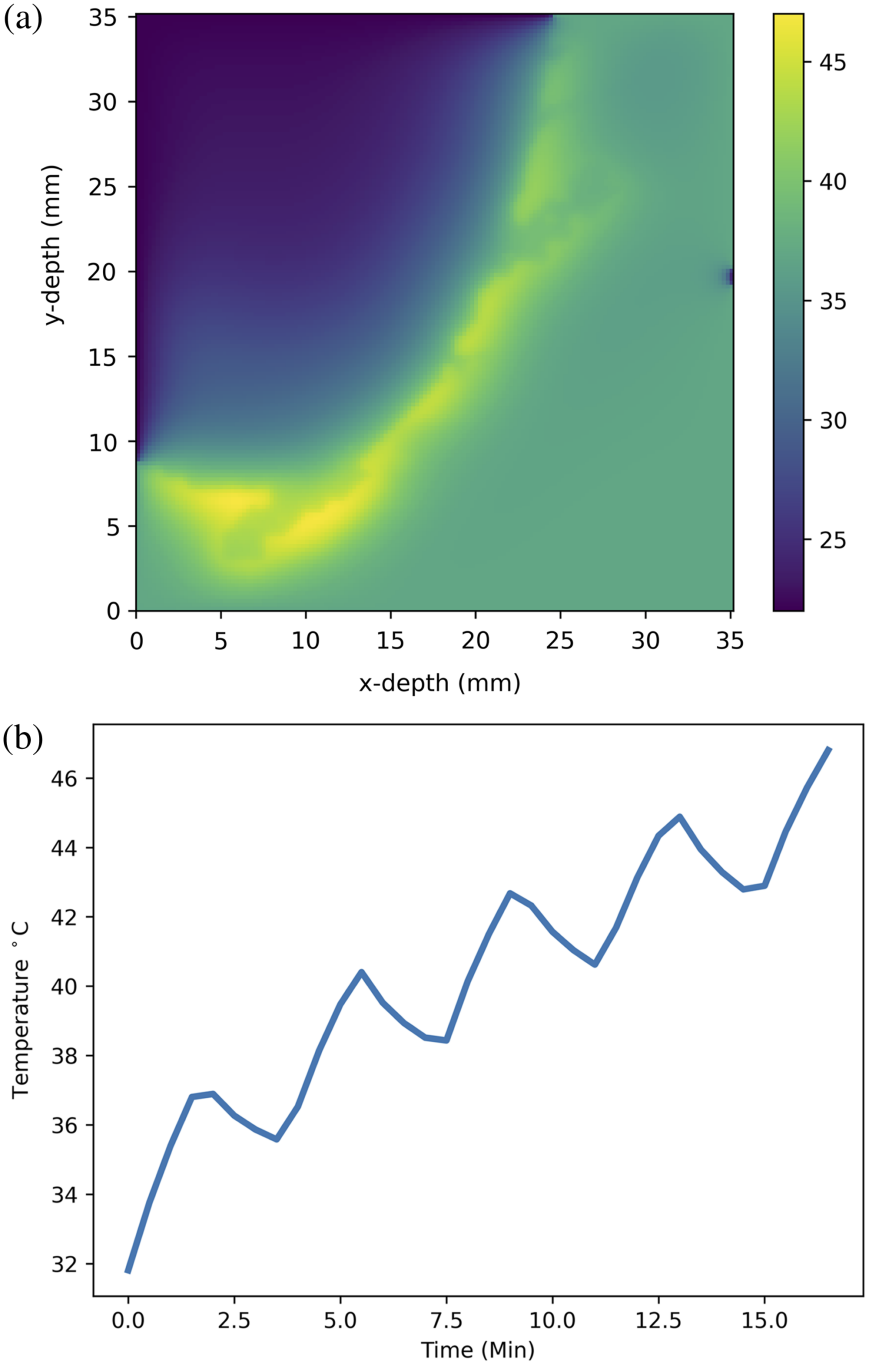
(a) Heat map showing the final temperature, after the standard protocol, of the grid slice of the resection cavity where the maximum temperature of 47°C is reached. The varying colors represent the temperature in °C as shown by the color bar to the right of the image. (b) Line plot showing the maximum tissue temperature reached over the full treatment time of 16.6 min for the standard protocol, reflecting the cooling effect of each fractionation.

The heat diffusion code was run for the standard protocol and the temperature changes throughout the subsection of the model recorded over the full treatment time. Figure 7(a) shows the final temperature of the slice where the maximum temperature is achieved.

The maximum temperature reached was also recorded throughout the treatment time and is plotted in Fig. 7(b), where it shows a maximum value of 47°C.

### 3.3 Parameter Concentration and Tissue Temperature with Depth

**Fig. 8:**
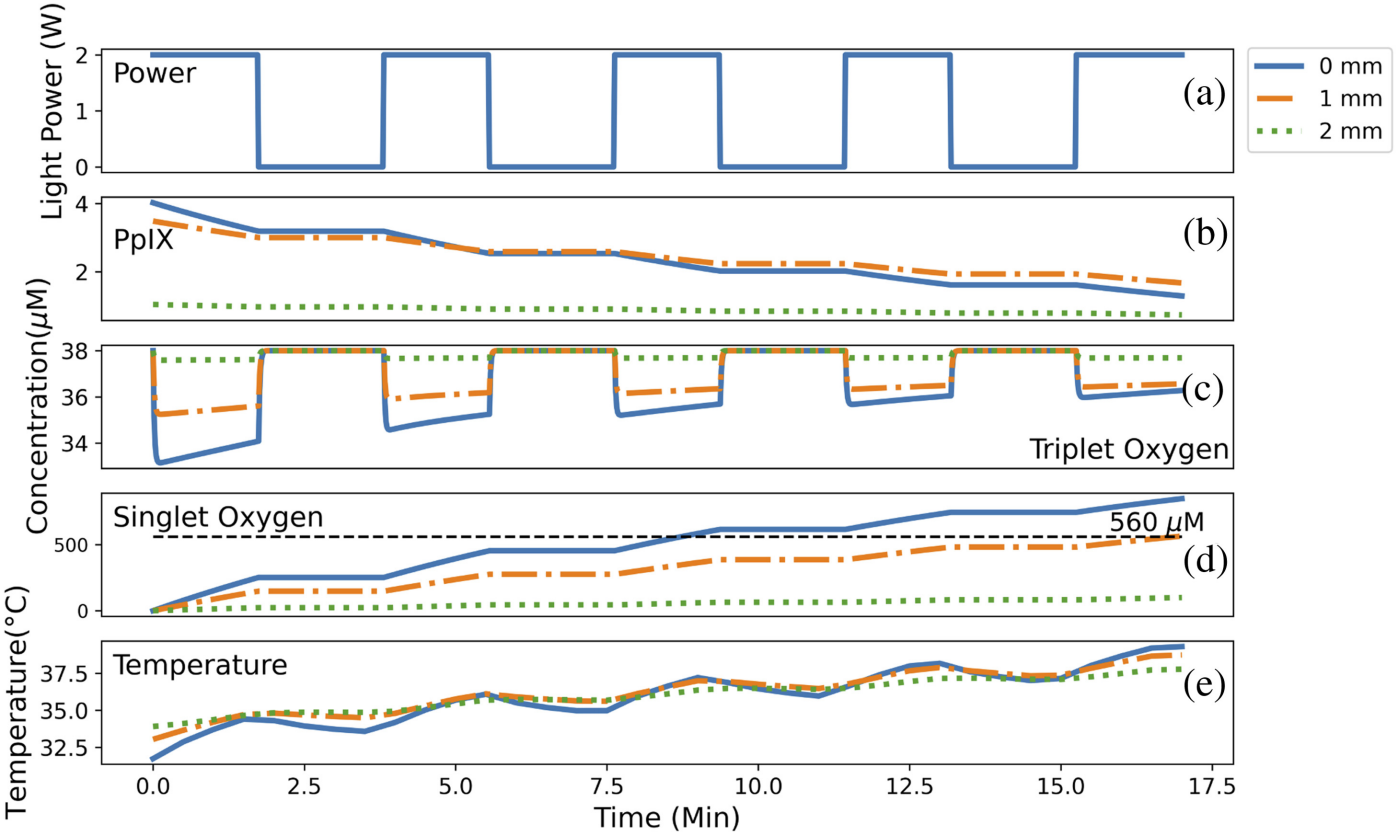
(a)–(e) Plots demonstrating the change in light power, PpIX, triplet oxygen and singlet oxygen concentration and tissue temperature over time at three different depths from the resection cavity wall along the x-line shown in Fig. 4(a). The initial PpIX concentration was 5  μM. There is very little PpIX at 2 mm as this is around the very edge of the tumor boundary (Fig. 5) where the tumor cell density is very low. The singlet oxygen plot demonstrates that, along this line, cell kill is only possible up to 1 mm using the standard protocol and the cell kill threshold concentration of 560  μM.

The concentrations of PpIX, cellular (triplet) oxygen and singlet oxygen over time for three different depths along the x-line indicated in Fig. 4(a) are plotted in Fig. 8. The top plot in Fig. 8 shows the total incident power over time using the standard 2 W fractionated source.

The percentage of GBM cells contained within a voxel decreases further away from the resection cavity walls, resulting in a decrease in the initial concentration of PpIX with depth. The PpIX concentration also decreases more rapidly over time at smaller depths from the wall, likely due to the larger light fluence there as shown in Fig. 5(a).

The third plot [Fig. 8(c)] shows cellular or triplet oxygen concentration. Here we can see that for smaller depths, the oxygen depletion is larger when the light is switched on. We can see from the second part of Eq. (7), that this faster decrease in cellular oxygen is again due to the larger fluence rate. However, we can also see that the oxygen recovery rate is faster at smaller depths, which, from the third part of Eq. (7), we can see is due to a difference in the cellular oxygen from the initial value, causing more oxygen to be added to the voxel per second. We can also see that the concentration that the oxygen is depleted by reduces over time for all depths, with the biggest reduction seen at the shallowest depth. This is likely due to the faster rate that the PpIX is being used up at shallower depths, causing the PDT process to slow down at a faster rate and resulting in less cellular oxygen being used up.

The fourth plot [Fig. 8(d)] shows the total concentration of singlet oxygen produced over time at each depth. As might be expected from looking at the previous two plots, the highest rate of singlet oxygen production is shown to be at the smallest depth, due to having the largest fluence rate. By looking at the cell kill threshold, marked by the horizontal black dashed line, we can also see that the standard treatment protocol is not causing GBM cell death beyond a depth of 1 mm along this line.

Finally, the last plot [Fig. 8(e)] shows the change in temperature over time at the three depths. At the beginning of the treatment, the smallest depth is coolest due to the faster loss of heat via conduction with the surface. However, this closer proximity to the surface means that smaller depths are subject to faster rates of heating from the PDT light source and by the end of the treatment, the smaller depth has the largest temperature.

### 3.4 Photosensitizer Concentration

**Fig. 9:**
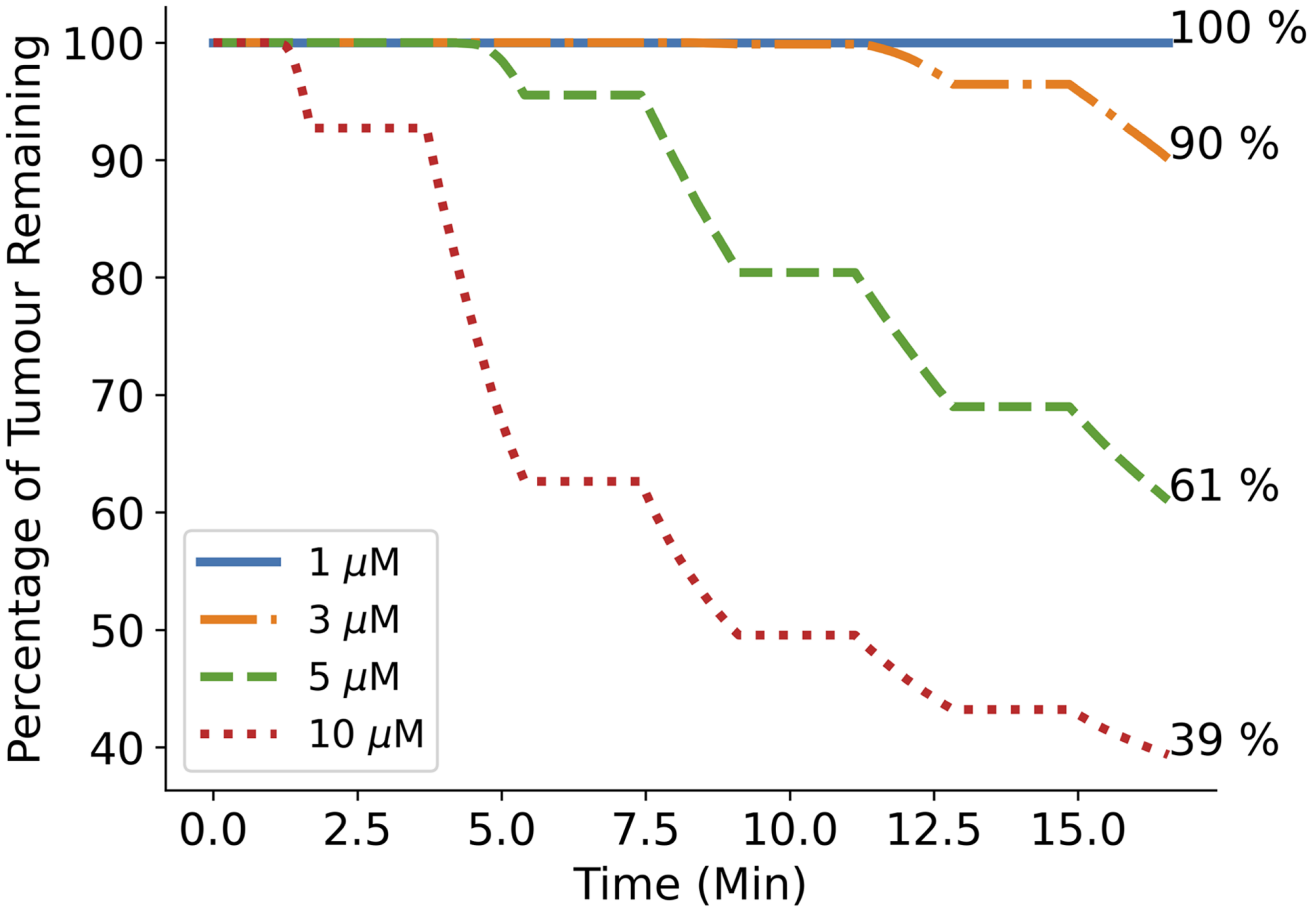
Plot showing the percentage of GBM cells remaining over the standard PDT treatment time with 2 W of light power and initial PpIX concentrations of 1  μM, 3  μM, 5  μM and 10  μM. While a concentration of 1  μM shows no evidence of cell kill, 5  μM results in 39% of GBM cells killed by the end of the treatment and this increases by a further 22% when the PpIX concentration is doubled to 10  μM.

The percentage of GBM cells remaining over the treatment time for initial PpIX concentrations of 1  μM, 3  μM, 5  μM, and 10  μM is plotted in Fig. 9. We can see that while no GBM cells are killed when using a concentration of 1  μM, 39% of the cells are killed with a concentration of 5  μM and 61% of cells are killed when doubling this to 10  μM. Assuming 95% of the tumor is removed during resection, this equates to 3.05% and 1.95% of the full tumor remaining after the standard protocol treatment with accumulated PpIX concentrations of 5  μM and 10  μM, respectively.

### 3.5 Oxygen Depletion

**Fig. 10:**
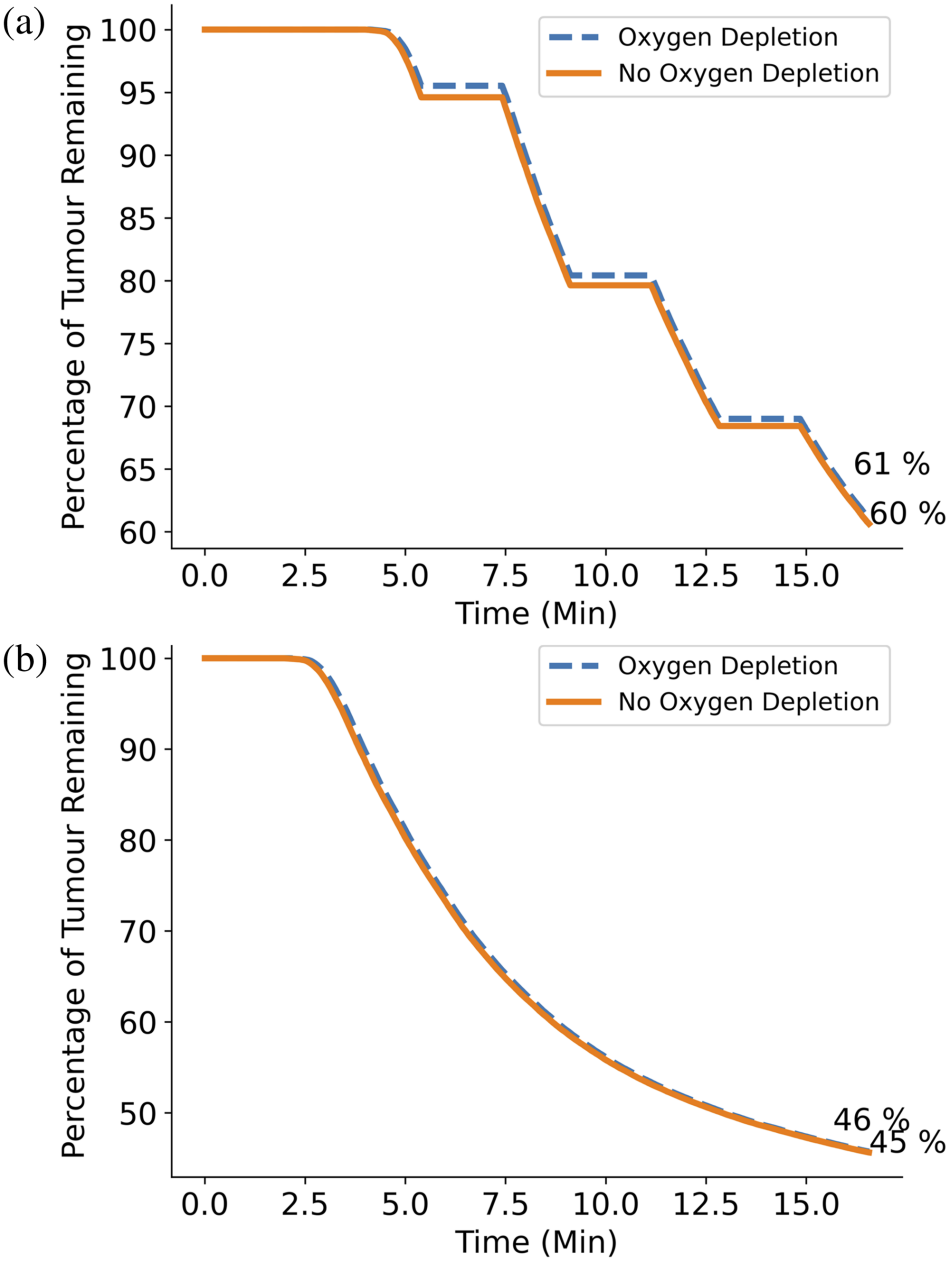
(a) Plot comparing percentage of GBM cells remaining over the standard PDT treatment time with 2 W light source and 5  μM initial PpIX concentration for the cases of normal cellular oxygen depletion (dashed) and no cellular oxygen depletion (solid). Only 1% difference is seen in GBM cell kill at the end of PDT treatment time. (b) Same as (a) except the light source fractionation is neglected. Similarly, very little difference is seen in overall GBM cell kill by the end of the treatment.

Figure 10 compares the percentage of GBM cells remaining over time using the standard treatment protocol, with and without cellular oxygen depletion and with a fractionated light source [Fig. 10(a)] and an unfractionated light source [Fig. 10(b)]. For both cases, little to no difference is seen in the percentage of remaining cells by the end of the treatment time when comparing the results of the simulations with and without oxygen depletion. Similarly, an equal percentage of GBM cells are left after the treatments with and without fractionation breaks. It seems, therefore, that oxygen depletion makes very little difference to the PDT treatment efficacy in this case. This is likely due to the oxygen recovery rate in brain tissue being sufficiently higher than the PDT oxygen usage rate, making oxygen levels recover fast enough that depletion does not pose an issue.^37^

### 3.6 Fractionation

**Fig. 11:**
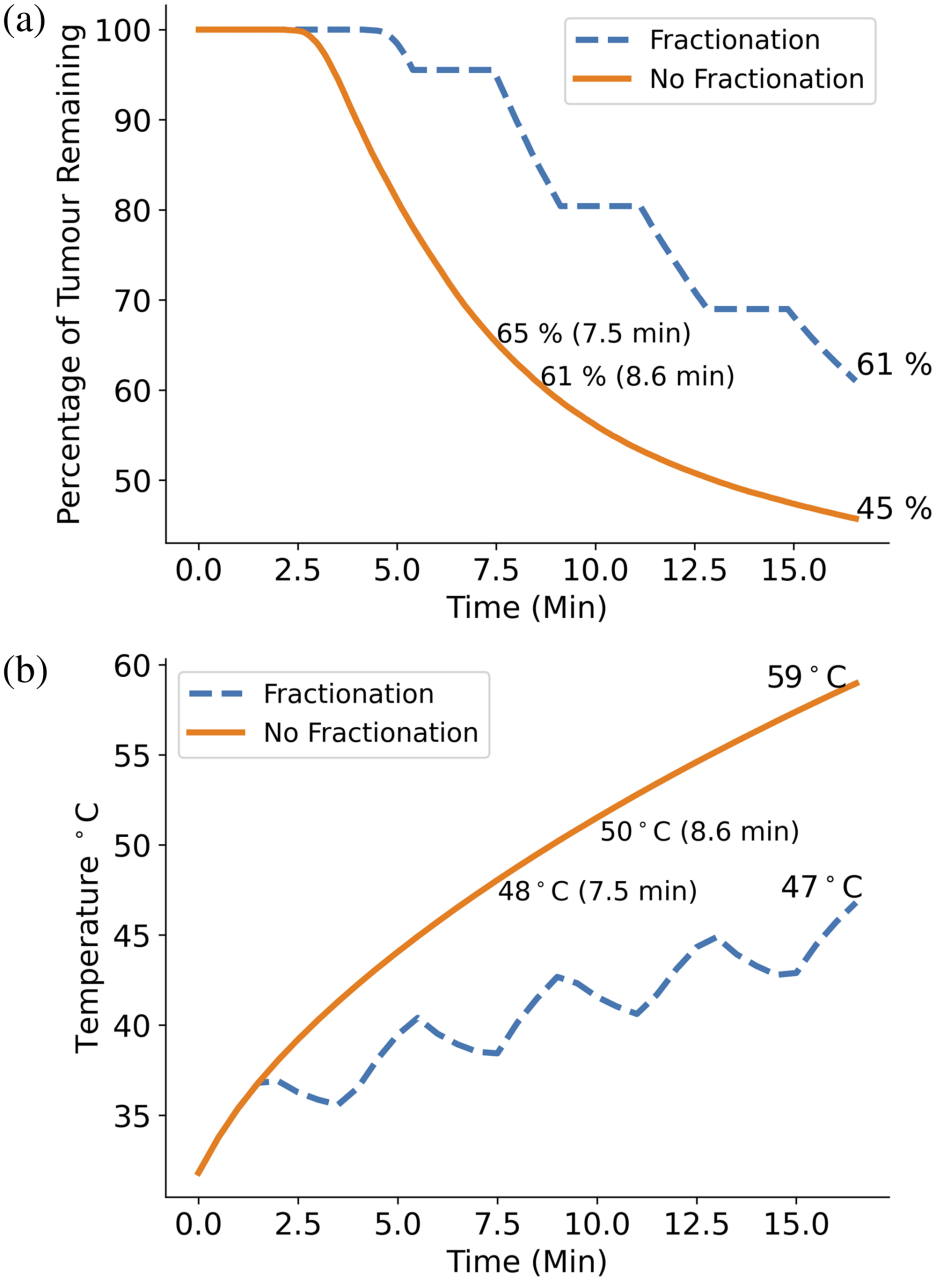
(a) Plot comparing percentage of GBM cells remaining over the standard PDT treatment time with 2 W light source and 5  μM initial PpIX concentration for the cases with standard fractionation of the PDT light source (four evenly spaced periods of 2 min where the light is switched off) (dashed) and where fractionation is neglected (solid). Using the unfractionated treatment light results in a GBM cell kill increase of 14% at the end of the treatment time. (b) Plot comparing the maximum tissue temperature over treatment time for the standard protocol with and without light fractionation. The standard protocol achieves a maximum temperature of 47°C. After an equivalent illumination time of 8.6 min, the non-fractionated treatment reaches a maximum temperature of 50°C, which then increases to 59°C after the remaining 8 min of treatment time. The plot also demonstrates that by shortening the unfractionated illumination time by 1.1 min to 7.6 min, the maximum temperature can remain a safe level while the overall GBM cell kill is only reduced by 4% compared to the fractionated, 16.6 min protocol.

As Fig. 10 demonstrates that oxygen depletion does not appear to affect treatment outcome, there may be benefit in choosing to keep the full treatment time including the extra time for fractionation breaks, but keep the light switched on for the full treatment. Figure 11(a) shows the percentage of remaining GBM cells over time when doing this compared to the standard, fractionated protocol. We can see that by allowing the light to stay on, GBM cell kill is increased by 16% by the end of the treatment.

However, from Fig. 11(b), we can see that the maximum tissue temperature reached by the non fractionated treatment is 59°C, 11°C above the damage threshold of 48°C.^2^ The maximum temperature at the end of the standard protocol however is just below this threshold at 47°C. After the same irradiation time of 8.6 min, the non fractionated treatment maximum temperature is 3°C higher than that of the standard protocol, suggesting that fractionation may be necessary to allow tissue cooling. However, reducing the unfractionated illumination time by just over 1 min to 7.5 min allows the maximum temperature to not exceed the damage threshold while only reducing the GBM cell kill by 4%, providing a much shorter treatment alternative.

### 3.7 Light Fluence

**Fig. 12:**
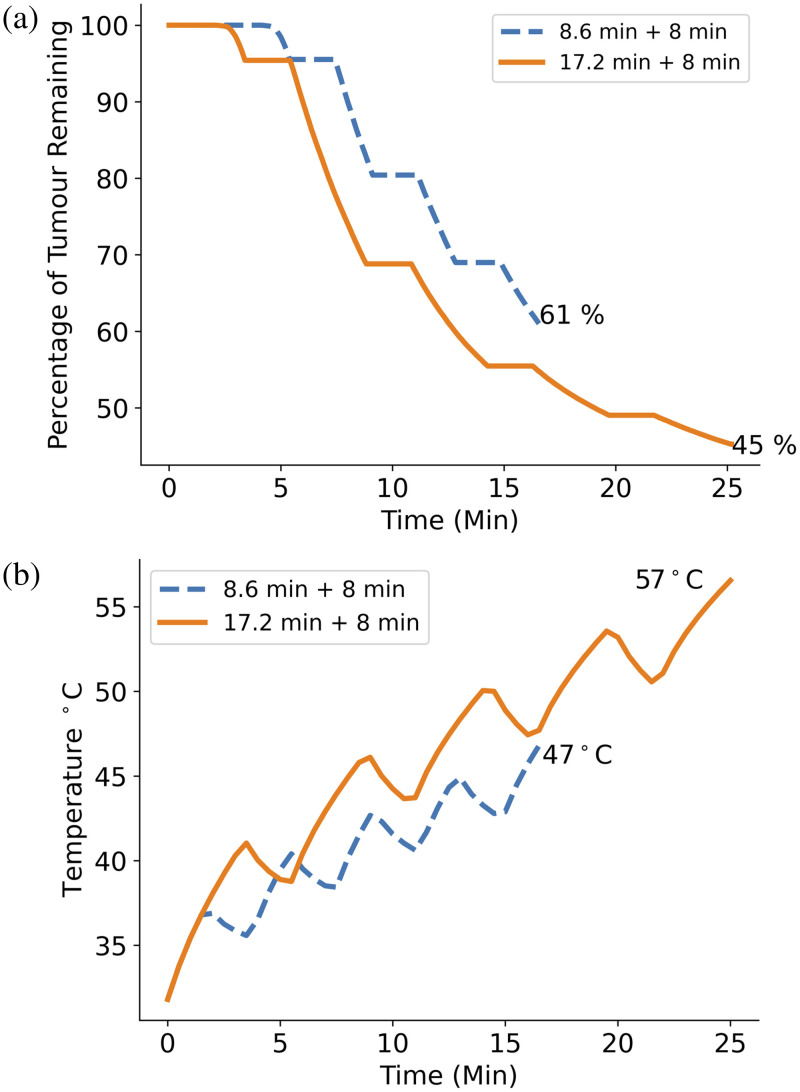
(a) Plot comparing percentage of GBM cells remaining with 2 W light source and 5  μM initial PpIX concentration for the cases of the standard treatment time (8.6 min + 8 min of fractionation breaks) (dashed) and double the treatment time (17.2 min plus 8 min of fractionation breaks) (solid). Doubling the standard treatment time results in a GBM cell kill increase of 16%. (b) Plot comparing the maximum tissue temperature over the treatment time for the standard protocol and the protocol with double treatment time. The maximum temperature seen at the end of the double treatment time protocol is 57°C, 9°C over the damage threshold of 48°C.

**Fig. 13:**
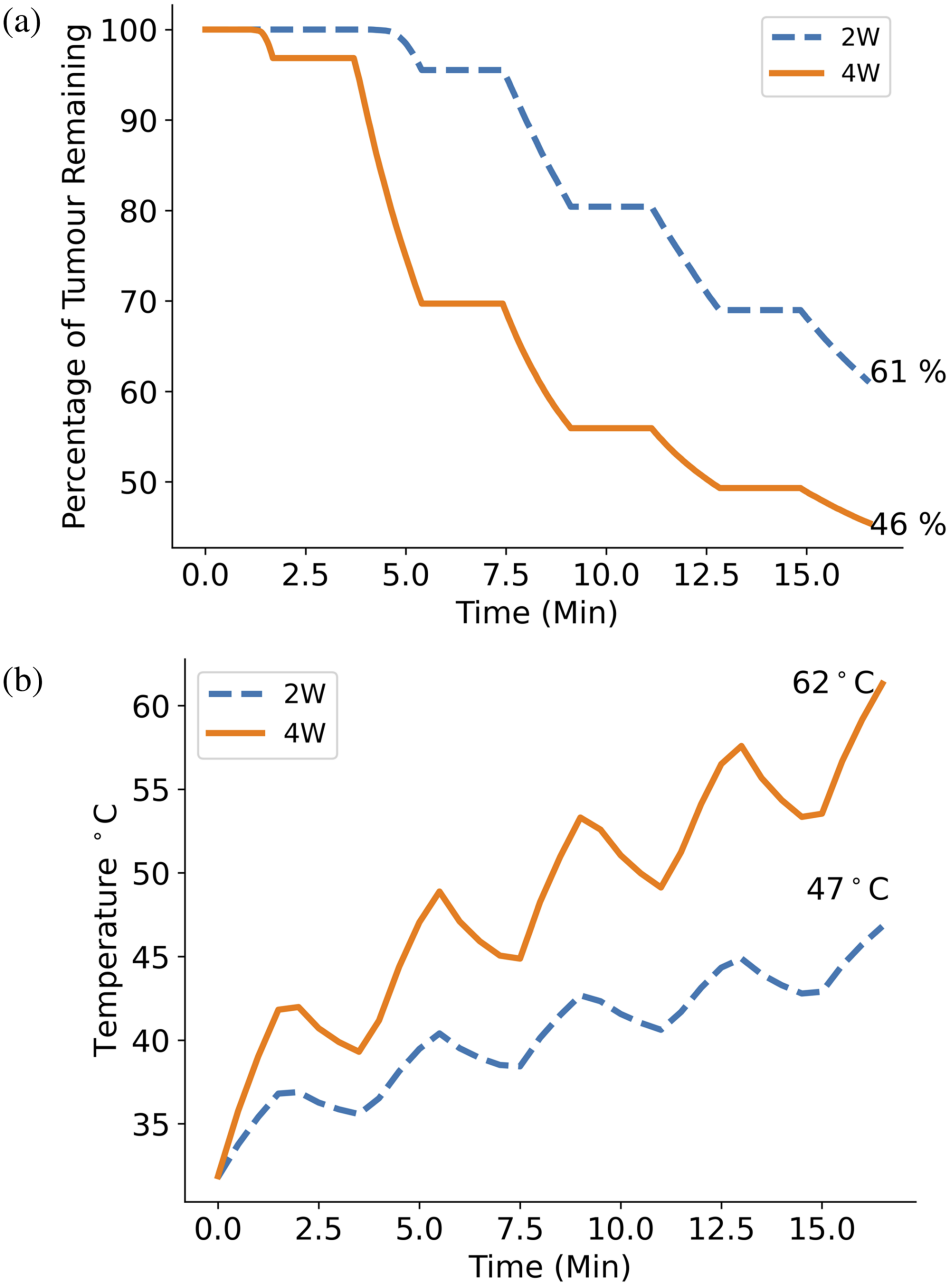
(a) Plot comparing percentage of GBM cells remaining using the standard treatment time and 5  μM initial PpIX concentration for the cases where the standard 2 W light source is used (dashed) and where a 4 W light source is used (solid). Doubling the standard treatment light power to 4 W results in a GBM cell kill increase of 15%. (b) Plot comparing the maximum tissue temperature over the treatment time for the standard protocol and the protocol with light power. The maximum temperature seen at the end of the double light power protocol is 62°C, 14°C over the damage threshold of 48°C.

The effect of doubling the total light fluence by either doubling treatment time or doubling light power compared to the standard protocol is shown in Figs. 12(a) and 13(a). In Fig. 12(a), it can be seen that the GBM cell kill increases by 16% when the treatment time is doubled from 8.6 min plus fractionation breaks to 17.2 min plus fractionation breaks. Similarly, in Fig. 13(a), it is shown that increasing the incident light power from 2 to 4 W results in a 15% cell kill increase. In both cases, the total light fluence is increased by the same amount, resulting in a similar cell kill increase; however, doubling the light power achieves this increase 8.6 min faster, which is highly relevant for clinical trial design. However, Figs. 12(b) and 13(b) show that the maximum temperature of the brain tissue in both cases exceeds the 48°C damage threshold, suggesting that doubling either treatment time or light power may not be a safe way of increasing treatment efficacy.

### 3.8 Cell Kill Threshold

**Fig. 14:**
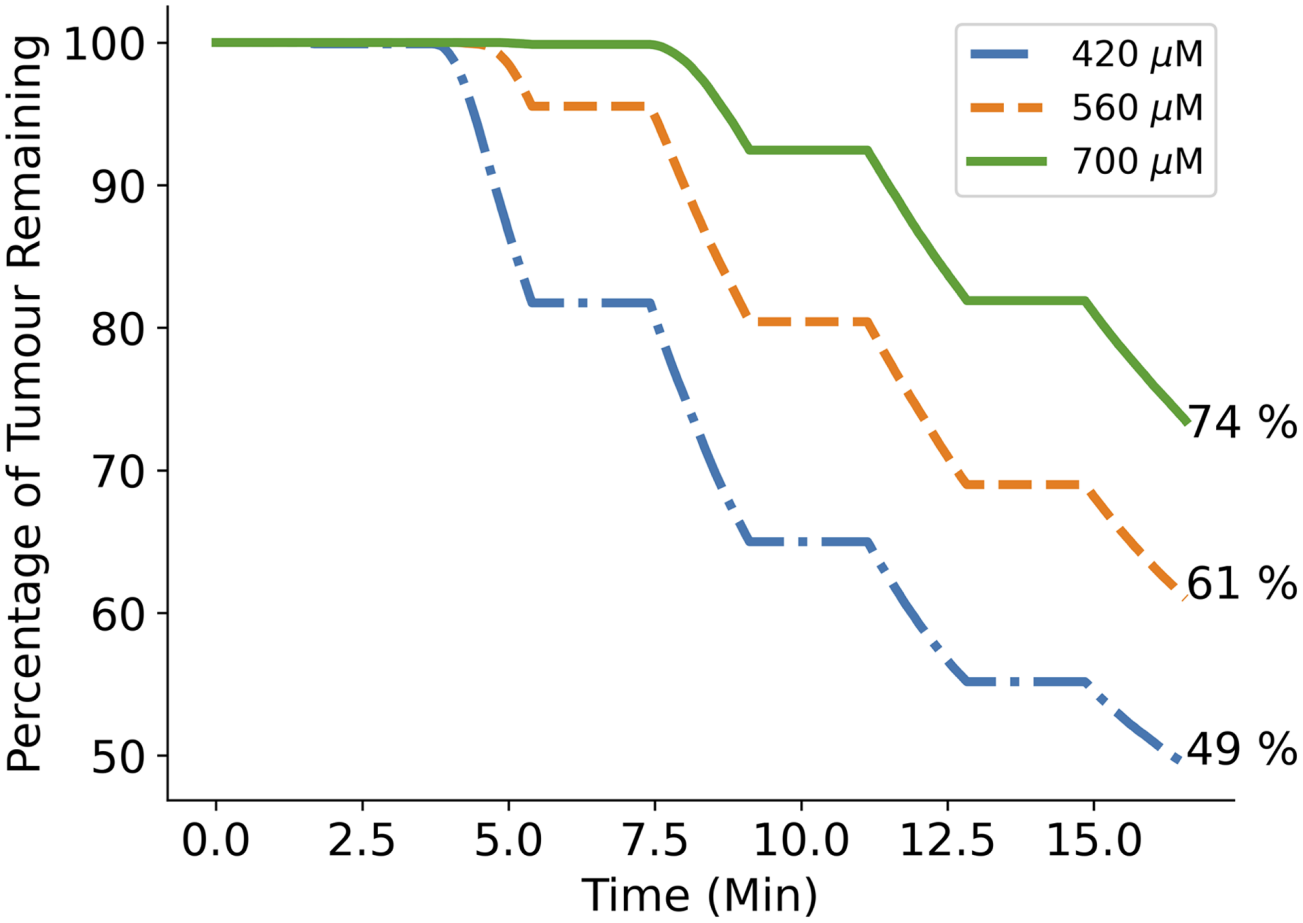
Effect of altering cell kill thresholds. Plot showing the percentage of GBM cells remaining over the standard PDT treatment time with 2 W of light power, 5  μM initial PpIX concentration and singlet oxygen concentration cell kill thresholds of the standard value (560  μM) and well as the standard plus 25% (700  μM) and the standard minus 25% (420  μM). Increasing the threshold to 700  μM results in a GBM cell kill reduction of 13%, whereas decreasing it results in a GBM cell kill increase of 12% compared to the standard value.

Finally, Fig. 14 shows the remaining GBM cells over time when using the standard protocol and varying the singlet oxygen concentration cell kill threshold by ±25% from the standard value of 560  μM. We can see that decreasing the threshold value to 420  μM increases cell kill by 12% while increasing the threshold to 700  μM decreases cell kill by 13%.

### 3.9 Optimal Protocol

**Fig. 15:**
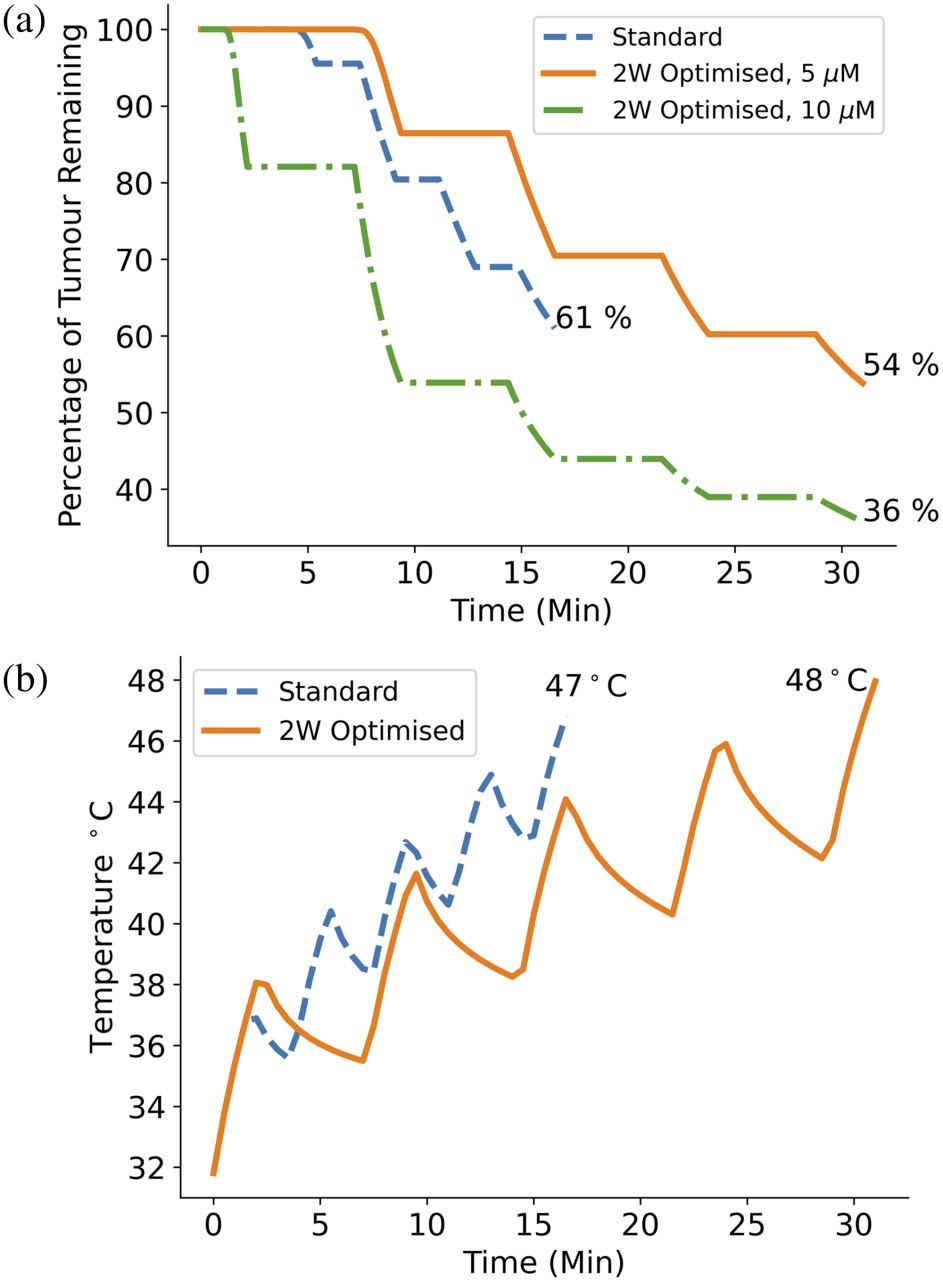
(a) Plot comparing the cell kill for the standard protocol and the optimized protocol using a 2 W light source. The optimized protocol involved using a longer treatment time of 11 min while extending the fractionation breaks to 5 min to allow sufficient tissue cooling. With the same initial concentration of PpIX (5  μM), the optimized protocol improved cell kill by 5%, which is improved by a further 18% when an initial concentration of 10  μM is used. However, further improvement within a 30 min time frame is limited by the maximum tissue temperature as shown in panel (b) where the optimized protocol has reached the damage threshold temperature of 48°C.

It was then investigated whether an “optimal” protocol could be found that increases the overall cell kill while keeping the tissue temperature at a safe level. To keep the treatment time at an acceptable level, it was decided that the total time, including fractionation breaks, should not exceed 30 min. Using the 2 W light source, a 5% improvement in cell kill [Fig. 15(a)] compared to the standard protocol was found by increasing the total irradiation time from 8.6 min to 11 min. This time increase resulted in the maximum temperature exceeding the damage threshold at 48°C; however by increasing the fractionation break time from 2 min to 5 min, the maximum temperature was brought back down to 48°C [Fig. 15(b)].

## 4 Discussion

The results above demonstrate that simple changes to the standard clinical trial protocol could be made to positively increase the efficacy of the treatment. Removing light fractionation and increasing the treatment time and light power are easy changes to incorporate. However, the results also demonstrate that while the INDYGO trials standard protocol keeps tissue temperatures within safe limits, increasing the light dose by incorporating these changes may increase the maximum temperature of the tissue to potentially damaging levels. The “optimized” protocol shown in Sec. 3.9 demonstrates a safe way that the treatment efficacy can be improved without overheating the tissue. The optimized protocol increases cell kill by about 5% compared to the standard protocol, the potential benefit of which should be weighted against the 14 min increase in total treatment time. It is possible that using tissue cooling methods such as irrigating the resection cavity with chilled saline or filling the balloon with chilled intralipid solution will help lower the maximum temperature reached, allowing for longer treatment times or larger light powers that can further improve cell kill. Saline irrigation already is used by Schipmann et al. during intraoperative PDT for high grade gliomas,^42^ which they find helps to reduce debris and blood clots within the resection cavity.

When examining the depth of cell kill in solid tumor (Sec. 3.1), we can see that most of the solid tumor sphere, used to represent a part of the tumor that was left undetected by the surgeon, is not removed by PDT. This suggests that PDT is a useful adjuvant treatment only when successful resection has taken place (>95% tumor removal).

When considering the study results, it can be seen from Fig. 9 that initial photosensitizer concentration makes the largest difference to the percentage of GBM cells killed, with a percentage increase of 22% when the concentration is doubled from 5  μM to 10  μM. It seems, therefore, that finding ways to improve photosensitizer uptake and accumulation will have a positive and prominent effect on the treatment outcome and is less likely to result in tissue damaging temperatures. Recent studies have shown promise when trying to increase tumoral PpIX concentrations using methods, such as utilizing nanoparticles for photosensitizer delivery^43^ and photobiomodulation.^44^ A study using ABCG2 transporter inhibitors to further inhibit the breakdown of PpIX has also shown promise by increasing PpIX concentrations *in vitro* in human glioma cell lines.^45^ However, while higher PpIX concentrations and light fluences appear to cause increased cell kill, a mouse study looking at the effect of PDT on blood brain barrier (BBB) permeability has suggested that PpIX doses above the standard 20  mg/kg and light fluences above $15\;J/{\mathrm{cm}}^{2}$ are associated with permanent damage to the BBB and brain tissues.^46^ The potential effect of any increases in PDT dose on the BBB, either through increasing the light fluence or the PpIX concentration, must then also be considered.^11^ Increasing PpIX uptake may also increase the potential for PDT damage to healthy tissue. It is possible for small concentrations of PpIX to accumulate in healthy tissues (although it should noted that PpIX selectivity within the brain is particularly high).^47^ Based on the results in figure, 9 concentrations of less than 1  μM should not cause cell kill. However, increasing tissue uptake of PpIX globally within the brain may result in larger accumulated PpIX concentrations in healthy tissue, increasing the potential for damage.

During the initial part of the INDYGO trial protocol, PpIX mediated FGS is used to aid the surgical tumor resection. ^13^ Due to the absorption spectrum of PpIX, the 420 nm light used for FGS will likely induce an initial PDT effect. However, this is assumed not to affect the overall results due to the low intensity of the light as well as the smaller penetration depth into tissue of 420 nm light, resulting in us studying only tissue that will either be surgically removed or killed later by the 635 nm light.

It should be noted that several assumptions were made within the simulation for simplicity and likely resulted in some sacrifice to the accuracy of results. For example, the PDT algorithm used assumes a Krogh cylinder model when describing oxygen transport.^31^^,^^32^ The Krogh model makes the assumption that oxygen is diffused into tissues radially from parallel cylindrical capillaries.^48^ While this model was relatively easy to incorporate into the MCRT simulation and provides relatively realistic results when modeling tissues with evenly spaced capillaries and homogeneous oxygen consumption, results are likely to be less accurate when modeling brain tissues, which contain complex capillary networks with uneven spacing and locationally variable oxygen consumption.^49^ Using a more complex vascular model would therefore lead to an improvement in result accuracy; however, computationally, it would be more expensive.

As discussed in Sec. 2.5.7, a fixed value for the singlet oxygen concentration cell kill threshold is used. It is very likely that the actual threshold value varies between tissue and photosensitizer types.^47^ It should then be noted that the work that defined 560  μM as the threshold value was using Photofrin as the photosensitizer and not PpIX.^39^ Unfortunately a measured value for the concentration of reacted singlet oxygen needed for cell kill when using PpIX does not yet exist in literature. It is also the case that several of the parameters (ξ and β) used within the PDT rate equations (see Table 2) for PpIX were assumed equal to those for Photofrin, also due to the fact that literature values do not yet exist for PpIX.^31^ It is for this reason that the results in Fig. 14 were produced, to allow an estimation of how the results may vary with a different cell kill threshold value. A great benefit of computational simulation is that as more accurate parameters become available, it is simple to update the results. The next stage of this work will likely focus on gaining a more accurate threshold value for PpIX while also further validating the model against clinical measurement results. Until then however, the threshold value measured when using Photofrin was assumed to be a good estimate. The results in Fig. 14 have also shown that a change in the threshold value affects results linearly, and so, while the actual cell kill percentages may not be fully relied upon, the simulation is a useful tool for comparing how changes to different parameters affect cell kill, relative to the standard protocol.

Finally, it should be noted that the numerical results obtained within this paper are an estimation and apply only to the brain model used. While qualitative results, such as methods to increase cell kill may be applicable to other brain and tumor geometries, quantitative results such as the specific percentage of GBM killed or the maximum temperature achieved will vary. The overall cell kill may also vary in reality due to other factors that are not considered such as an immune response^2^ and due to the fact that several of the parameters used had to be estimated.

## 5 Conclusion

A Monte Carlo simulation of intraoperative PDT for the treatment of GBM is presented. The simulation incorporates light fluence calculations as well as photosensitizer concentration, oxygen depletion and tissue temperature. The impact of treatment parameters, such as light power, photsensitizer concentration and treatment time on GBM cell kill was investigated with the outcome calculated based on the concentration of singlet oxygen produced. The results gained within this work have direct potential clinical benefit as evidence within literature shows that maximizing tumor removal leads to improvement in treatment outcome. The simulation results suggest that the current standard protocol of the INDYGO trial manages to obtain a good level of GBM cell kill within a reasonably short treatment time while keeping the temperature of the brain tissue at a safe level. Higher light fluences lead to the tissue temperature exceeding the maximum safe level or require significantly larger treatment time to allow tissue cooling breaks, improving the cell kill by around 5%, a result that, with further model validation, could provide some clinical benefit if the longer treatment time is acceptable. A short treatment time seems to be favored and so the standard protocol appears to be the optimal current solution. Higher initial PpIX concentrations lead to a large increase in the percentage of GBM cells killed, suggesting that finding ways to improve photsensitizer uptake will lead to a positive improvement in the treatment efficacy.

## Data Availability

The MCRT code used within this work is available at https://gitlab.com/Loufin101. The data presented in this article are publicly available in Zenodo at https://doi.org/10.5281/zenodo.8302340.
