# Supplementary material for: Simulating photodynamic therapy for the treatment of glioblastoma using Monte Carlo radiative transport
Source: J Biomed Opt. 2024 Feb 6;29(2):025001. doi: 10.1117/1.JBO.29.2.025001 (PMC10846422; doi:10.1117/1.JBO.29.2.025001)
Supplement: Supplementary file 1 [file JBO_029_025001_SD001.pdf]

Supplementary Material

1 Validation

1.1 Fluence rate

To validate the calculated fluence rate within the MCRT code, measurement results from an MCRT code developed by Jacques et al.[29] were reproduced. They used their code to develop equations (equation 1))for the fluence rate at depth z into a skin model, based on the measured optical properties of rat skin.

$$\Psi(z) = \Psi_0(C_1 e^{(\frac{-k_1 z}{\delta})} - C_2 e^{(\frac{-k_2 z}{\delta})}) \tag{1}$$

Here Ψ_0 is the irradiance of the light $(\mathrm{W/cm^2})$ at the skins surface surface, C_1, C_2, C_3, k_1, k_2 and k_3 are the wavelength dependant fitting parameters described in table 1. δ is the optical penetration depth described by 2. Values for δ as well as the absorption coefficients (μ_a) and scattering coefficients (μ_s) are also found in table 1.

$$\delta = \frac{1}{\sqrt{3\mu_a(\mu_a + \mu_s(1-g))}} \tag{2}$$

λ (nm)	C_1	C_2	k_1	k_2	μ_a ($\mathrm{cm^{-1}}$)	μ_s ($\mathrm{cm^{-1}}$)	δ (cm)
420	5.76	1.31	1.00	10.2	1.8	82	0.047
630	6.27	1.18	1.00	14.4	0.23	21	0.261

Table 1 Optical properties of rat skin and fitting parameters used in equation 1[29].

To reproduce equation 1 within the MCRT code, a 2 cm x 2 cm x 2 cm voxel grid was used with 100 x 100 x 100 voxel and the optical properties from table 1 were applied. The light was set

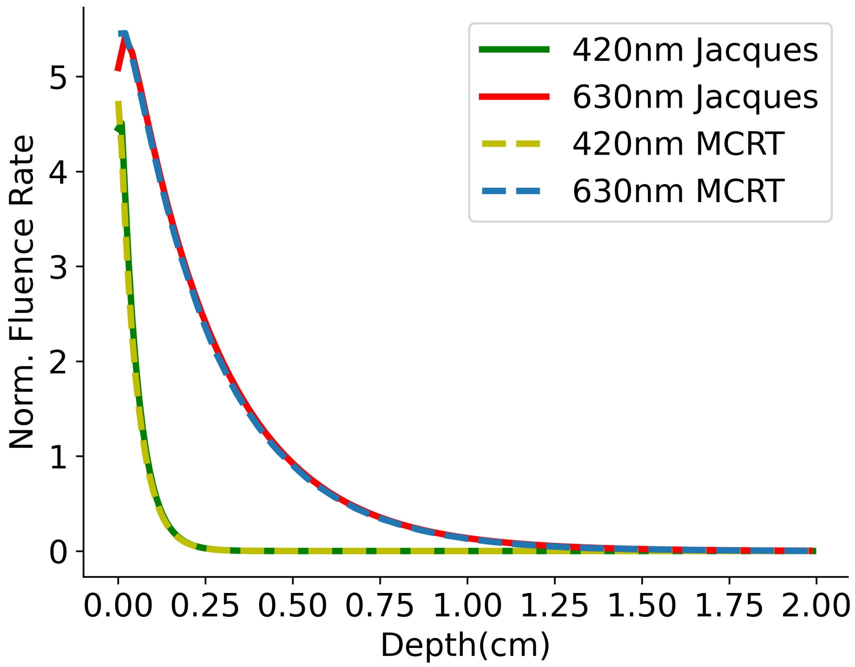

Fig 1 Plot comparing the normalised fluence rates calculated using equation 1 developed by Jacques et al.[29] to the MCRT simulation results . The plot shows results for both 420 nm and 630 nm light, with the MCRT and Jacques values matching well.

to be uniformly distributed over the top of the grid with an irradiance of 0.1 $\mathrm{W/cm^2}$.

Figure 1 compares the normalised fluence rates with depth into the grid obtained by the MCRT code to the results from the developed function (equation 1) for both 630 nm and 420 nm.

1.2 Oxygen diffusion and photobleaching

To validate the PDT part of the simulation, a simulation was setup to reproduce PDT measurements taken by Wang *et al.* during their work to develop the PDT algorithm[31]. The measurements from their 'mouse 3' model were reproduced. Within the code, a voxel grid containing 100 x 100 x 100 voxels and with dimension 2 cm x 2 cm x 2 cm was used. The optical properties used were the absorption coefficient ($\mu_a = 1.51\mathrm{cm}^{-1}$) and the reduced scattering coefficient ($\mu_s' = 12.29\mathrm{cm}^{-1}$)

and were measured by Wang *et al.* at 630 nm. An initial photosensitiser concentration of 3.7 μM was also applied.

During the measurements, Wang *et al.* used a linear light source with a power of 33 mW/cm and a total delivered energy of 88 J/cm. This corresponds to a treatment time of 44.4 minutes. They then used a finite element method (FEM) code to simulate the fluence rate. To simulate this within the MCRT code, a line of isotropic point sources were used as the linear source which was placed in the centre of the grid to a depth of 1 cm and set to emit the correct power. The code was then run in a loop, with each loop corresponding to 1 second of measurement time until the 44.4 minutes were complete.

The simulated fluence rate, photosensitiser concentration, triplet oxygen concentration and singlet oxygen concentration at the end of the measurement time were plotted along the direction perpendicular to the linear source and compared to Wang *et als.* results in figures 2 to 5.

1.3 Temperature

The code used for the temperature simulation was validated against an analytical case by McMillan *et al.*[22]

1.4 Cell kill

Unfortunately the simulated rate of cell kill could not be validated against any measurements. Cell kill is not something that can be easily measured *in vivo* and so the current simulation can only act as an estimate. This is something we hope to improve upon in future work by exploring both the

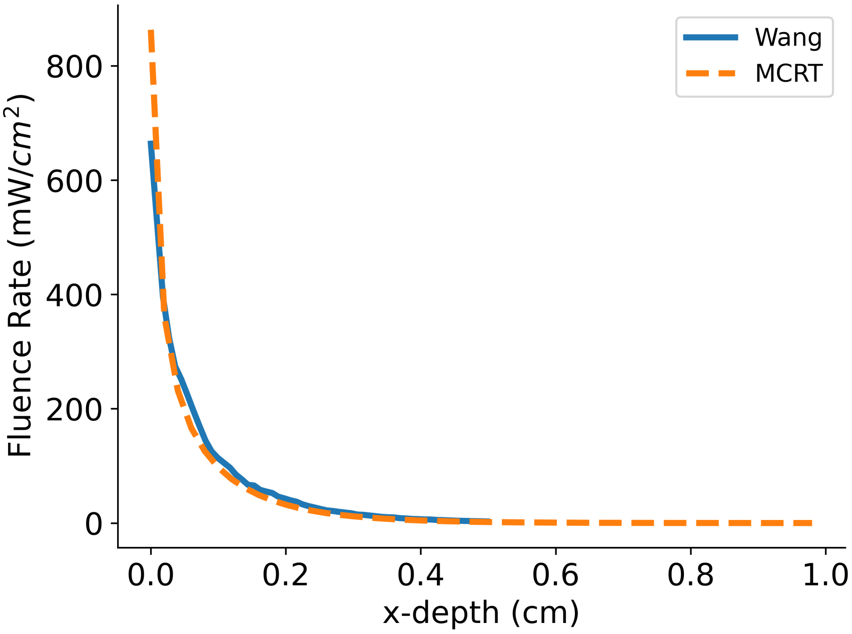

Fig 2 Plot comparing the calculated fluence rates from Wang *et als.* FEM simulation for mouse 3 and the produced MCRT simulation. Light power was 33mW/cm with a wavelength of 630 nm and a total measurement time of 44.4 minutes.

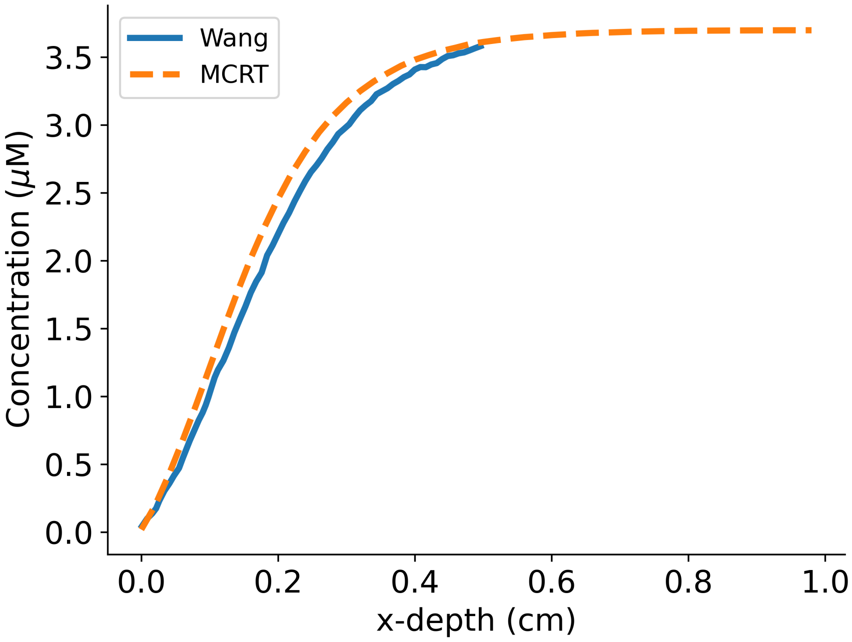

Fig 3 Plot comparing the photosensitiser concentration obtained by Wang *et als.* and the produced MCRT simulation.

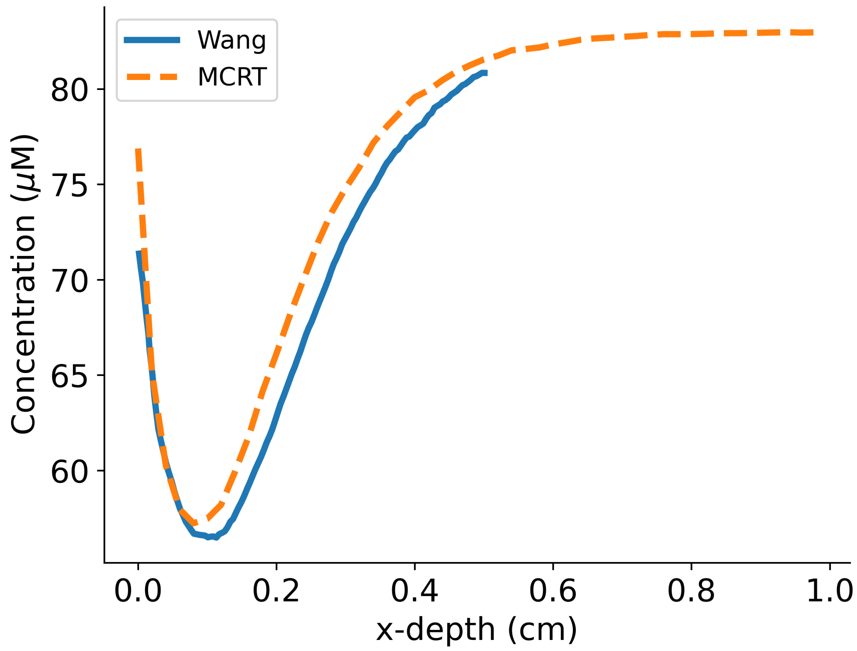

Fig 4 Plot comparing the triplet oxygen concentration obtained by Wang *et als.* and the produced MCRT simulation.

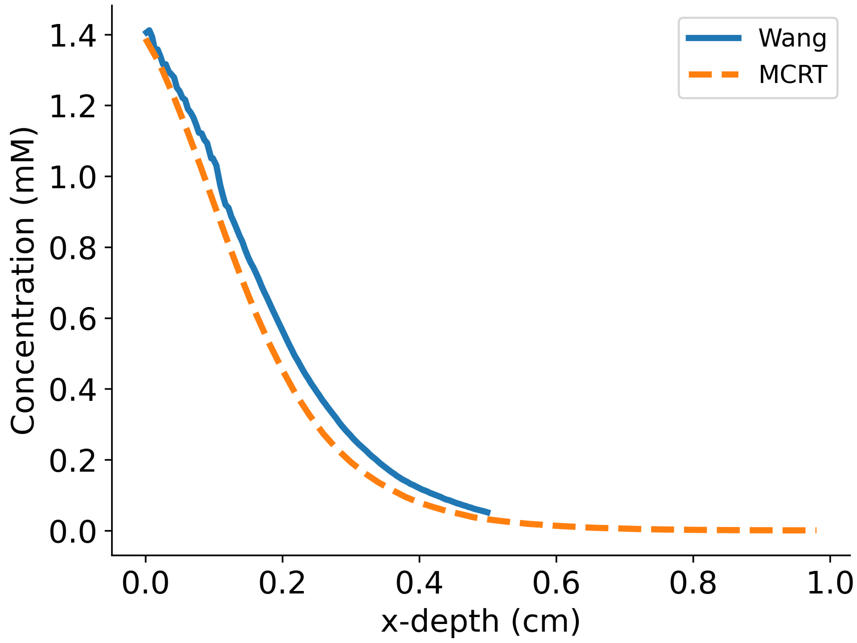

Fig 5 Plot comparing the singlet oxygen concentration obtained by Wang *et als.* and the produced MCRT simulation.

cell kill threshold and the rate of cell kill in more clinical detail.
